# Virus infections and cancers: from mechanisms to therapeutics

**DOI:** 10.1186/s43556-026-00485-6

**Published:** 2026-07-08

**Authors:** Yu Li, Wenjie Yu, Jiaxin Yun, Jingqian Wang, Yakun Liu, Huancheng Su, Hongrui Guo, Jiaolin Yang, Yaru Yan, Xiaoliang Yan, Sanyuan Zhang, Hailan Yang, Zhe Wang

**Affiliations:** 1 Department of the First Clinical Medical College, Shanxi Medical University, Taiyuan, 030001 China; 2 Department of Gynecology, First Hospital of Shanxi Medical University, Taiyuan, 030001 China; 3 Department of Psychiatry, First Hospital of Shanxi Medical University, Taiyuan, 030001 China; 4 Department of Thoracic Surgery, First Hospital of Shanxi Medical University, Taiyuan, 030001 China; 5 Department of Obstetrics, First Hospital of Shanxi Medical University, Taiyuan, 030001 China

**Keywords:** Carcinogenic viruses, Tumor microenvironment, Epigenetics, Biomarkers, Precision therapy

## Abstract

Viral infections are a major contributor to global cancer incidence and mortality. However, integrative reviews that connect viral classification, carcinogenic mechanisms, tumor microenvironment remodeling, and translational strategies remain limited. This review summarizes the classification and epidemiological characteristics of major oncogenic viruses, including human papillomavirus (HPV), Epstein-Barr virus (EBV), hepatitis B virus (HBV), hepatitis C virus (HCV), Merkel cell polyomavirus (MCPyV), human T-lymphotropic virus type 1 (HTLV-1), Kaposi’s sarcoma-associated herpesvirus (KSHV), and human immunodeficiency virus (HIV), as well as emerging viruses such as severe acute respiratory syndrome coronavirus 2 (SARS-CoV-2). We then discuss the molecular basis of virus-associated carcinogenesis, including viral oncogenes, viral DNA integration, epigenetic remodeling, aberrant host signaling, and metabolic dysregulation. We further examine how chronic inflammation and fibrosis create oncogenic niches within the tumor microenvironment (TME), how viruses promote tumor progression through immune evasion and immune exhaustion, and how infected cells interact with stromal and immune components of the TME. At the preventive and therapeutic levels, we discuss antiviral therapies, vaccines, biomarker-based precision diagnostics, and prognostic strategies, with particular attention to the synergistic potential of emerging therapeutic approaches such as immune checkpoint inhibitors (ICIs), CAR-T therapy, and oncolytic viruses (OVs). Finally, we highlight how multi-omics approaches, single-cell transcriptomics, spatial transcriptomics, organoid models, and artificial intelligence can advance mechanistic studies and translational innovation in virus-associated cancers. Overall, this review provides an integrated framework for understanding, preventing, and treating virus-associated tumorigenesis.

## Introduction

Research on virus-cancer links dates back to the early twentieth century, when the hypothesis of virus-induced cancer first emerged [1]. Although initially controversial, this concept laid the foundation for modern tumor virology and was ultimately recognized by the 1966 Nobel Prize awarded to Peyton Rous. It is now well established that approximately 15%−20% of human cancers are directly attributable to viral infections [2]. Among the best-characterized oncogenic viruses are HPV, EBV, HBV, and HCV, which drive tumor initiation, progression, and, in some settings, metastasis through persistent infection and long-term reprogramming of host cells. In 2022, HBV/HCV-related liver cancer accounted for approximately 870,000 new cases and 760,000 deaths worldwide [3], while HPV-related cervical cancer caused about 662,000 new cases and 349,000 deaths [4]. In parallel, growing attention has turned to emerging viruses such as severe acute respiratory syndrome coronavirus 2 (SARS-CoV-2), whose indirect effects on chronic inflammation, immune dysregulation, and cancer outcomes have broadened the scope of viral oncology research [5].

Despite major advances in vaccines, antiviral therapy, and mechanistic studies, important challenges remain. Virus-associated cancers differ substantially in tissue tropism, latency programs, immune interactions, and clinical behavior. For example, infection-related tumors account for about 12% of all new cancer cases worldwide when other pathogens such as Helicobacter pylori are also considered [6]. The burden and trajectory of virus-associated cancers are therefore highly context dependent: HPV vaccination and screening have markedly reduced cervical precancer in some populations, with a 79.5% decline in cumulative CIN2 + incidence among U.S. women aged 20–24 between 2008 and 2022 [7], whereas HBV/HCV-associated hepatocellular carcinoma remains a major global challenge, with projected liver cancer cases reaching 1.52 million and deaths 1.37 million by 2050 without stronger intervention [3]. These contrasts underscore the need for a review that moves beyond virus-by-virus description and instead integrates common carcinogenic principles with clinically relevant differences across viruses.

In this review, we synthesize current knowledge on both classical and emerging oncogenic viruses from a unified framework. We first summarize viral classification, epidemiology, and the potential carcinogenic implications of emerging viral threats. We then examine the major mechanisms of viral tumorigenesis, including genome integration, epigenetic remodeling, signaling dysregulation, metabolic rewiring, and stage-specific roles in tumor initiation, promotion, metastasis, and therapeutic resistance. Next, we discuss how persistent infection reshapes the tumor microenvironment through chronic inflammation, fibrosis, immune evasion, immune exhaustion, and intercellular communication. Finally, we highlight progress in prevention, diagnosis, and treatment, with emphasis on antiviral therapy, immunotherapy, precision diagnostics, and emerging technologies such as multi-omics, organoids, single-cell and spatial transcriptomics, and artificial intelligence. By combining mechanistic synthesis with translational perspective, this review aims to provide a clearer conceptual map of virus-associated cancers and to identify directions for future research and clinical management.

## Classification and epidemiology of virus-induced cancers

Viral infections constitute a major component of the global cancer burden. Rather than acting as isolated pathogens, oncogenic viruses influence carcinogenesis through a combination of persistent infection, direct oncogenic activity, immune modulation, chronic inflammation, and long-term tissue remodeling. It is estimated that approximately one-fifth of human cancers are attributable to infectious causes, with viruses accounting for a substantial proportion of this burden [8]. Understanding how these viruses are distributed, which cancers they are associated with, and how their biologic behavior differs across populations is essential not only for prevention and early detection, but also for building a coherent framework for virus-associated oncology.

Importantly, the epidemiology of virus-induced cancers is not uniform. Different oncogenic viruses exhibit distinct tissue tropisms, transmission routes, latency patterns, and opportunities for prevention. As a result, the global burden of virus-associated malignancies is shaped by geography, healthcare access, vaccination coverage, co-infection status, and underlying host susceptibility. In this chapter, we summarize the global burden of virus-induced cancers, outline the clinically most relevant oncogenic viruses, and discuss the emerging question of whether newly recognized viral threats may also contribute to cancer risk.

### Global burden of virus-induced cancers

Virus-induced cancers remain a major and persistent challenge to global public health. Approximately 15%−20% of cancer cases worldwide are estimated to be directly attributable to chronic viral infections [9, 10]. However, this burden is distributed unevenly across geographic regions and populations, reflecting differences in viral prevalence, host susceptibility, healthcare systems, vaccination programs, and screening infrastructure.

**Fig. 1:**
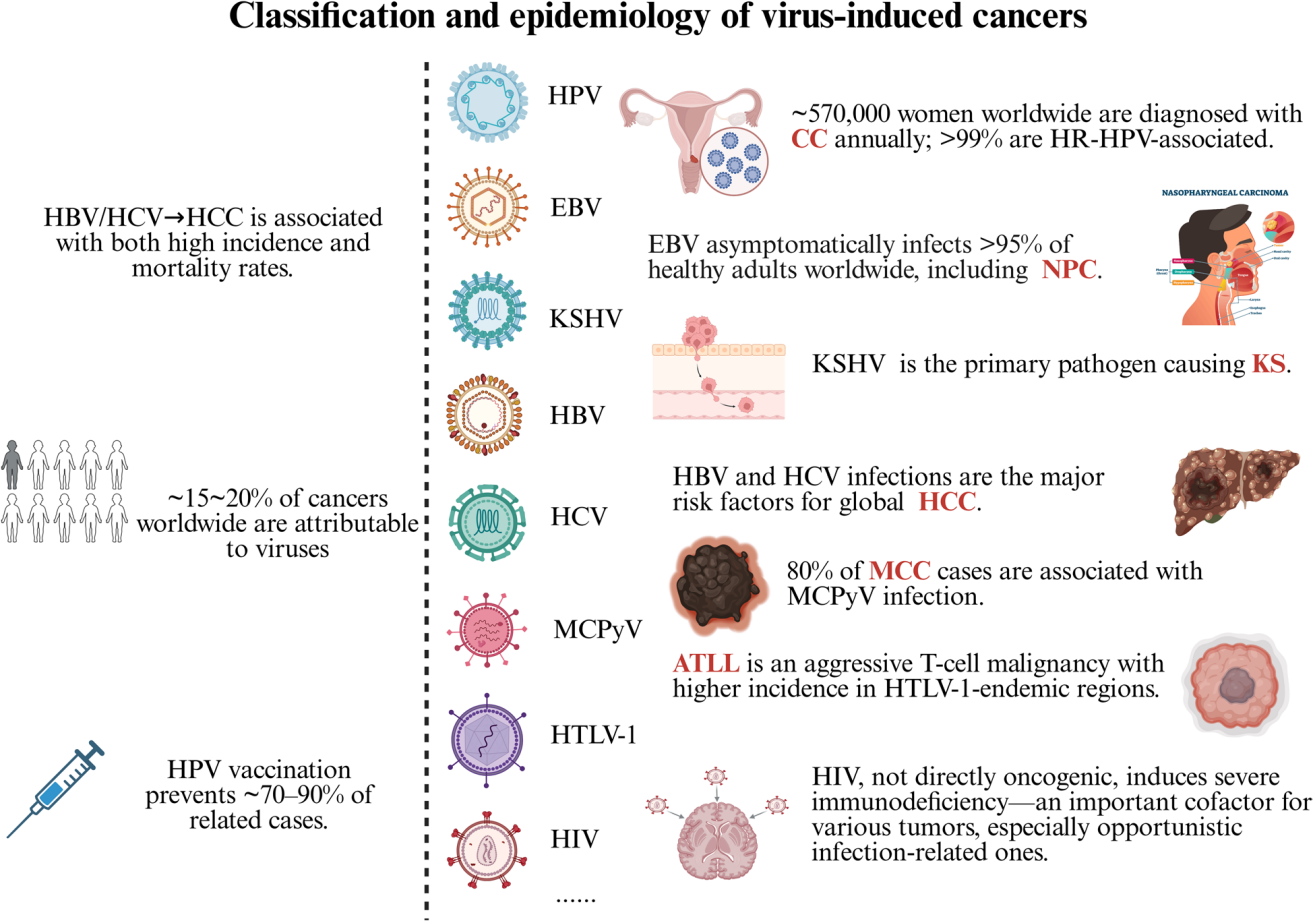
Classification and Epidemiology of Virus-Induced Cancers. (Left) Epidemiological characteristics of virus-induced cancers: Globally, approximately 15%–20% of cancers are attributable to viral infections, with the most significant burden being hepatocellular carcinoma associated with hepatitis B virus and hepatitis C virus. Nevertheless, early vaccine interventions can effectively control disease progression; for instance, HPV vaccines prevent approximately 70%–90% of related cases. (Right) Classification of Virus-Induced Cancers: HPV is the primary causative agent of cervical cancer, with over 99% of the approximately 570,000 new cases annually worldwide linked to high-risk HPV infection; EBV has a prevalence exceeding 95% in healthy adults and is strongly associated with malignancies such as nasopharyngeal carcinoma (NPC); KSHV is the primary pathogen of Kaposi's sarcoma (KS); HBV and HCV collectively constitute major risk factors for HCC; MCPyV is associated with approximately 80% of Merkel cell carcinoma (MCC) cases; HTLV-1 can cause adult T-cell leukemia/lymphoma (ALT) in endemic regions. Furthermore, while HIV does not directly cause cancer, it significantly increases the risk of various opportunistic tumors by inducing immune deficiency, making it an important co-carcinogenic factor

In regions such as sub-Saharan Africa, Southeast Asia, and East Asia, where chronic HBV and HCV infection remains common, hepatocellular carcinoma (HCC) occurs at rates substantially above the global average [11]. By contrast, in many high-income regions—including Western and Northern Europe, North America, and Australia—the incidence of HPV-associated malignancies, particularly cervical and oropharyngeal cancers, remains relatively prominent despite improvements in prevention and screening [12, 13]. Furthermore, a close association between EBV and the pathogenesis of nasopharyngeal carcinoma, gastric cancer, and a spectrum of lymphomas has been established. The epidemiological distribution of these EBV-driven malignancies exhibits pronounced geographical predilections and is further modulated by underlying genetic susceptibilities. (Fig. 1).

The impact of virus-associated cancers extends well beyond incidence and mortality statistics. These malignancies impose a substantial burden on healthcare systems, generate major economic costs, and frequently affect populations with unequal access to preventive care and early diagnosis [8]. This burden underscores the need for enhanced viral prevention and control strategies on a global scale.

### Oncoviruses of clinical relevance

Currently, seven viruses have been formally classified as human carcinogens by the International Agency for Research on Cancer (IARC) [8, 9]. These seven viruses include: HPV, EBV, KSHV, HBV, HCV, MCPyV and HTLV-1. Beyond these, while HIV itself is not a direct carcinogen, the severe immune deficiency it causes serves as a significant co-factor in the development of various tumors, particularly cancers associated with opportunistic infections. Clinically, these viruses are best understood not simply as a list of pathogens, but as distinct models of virus-associated carcinogenesis, each highlighting a different route by which persistent infection can promote malignant transformation.

#### Human Papillomavirus (HPV)

Among the most prevalent oncogenic viruses, HPV is transmitted primarily through sexual contact. The highest burdens of HPV infection are observed in Asia, Africa, and the Americas. Collectively, these regions account for 54%, 19%, and 16% of incident infections worldwide, respectively [14]. Worldwide, cervical cancer is diagnosed in over 570,000 women each year, and more than 99% of these cases are attributed to persistent infection with high-risk HPV genotypes—most notably HPV16 and HPV18 [15]. HPV is also implicated in vulvar, vaginal, anal, penile, and a subset of oropharyngeal and head and neck squamous cell carcinomas [12, 13].

The clinical importance of HPV lies not only in its broad cancer spectrum, but also in the fact that it represents one of the clearest examples of a malignancy preventable through organized public health intervention. The inverse association between cervical cancer incidence and national income largely reflects differences in access to vaccination, screening, and treatment infrastructure [8, 16]. High-coverage HPV vaccination is estimated to prevent 70%–90% of attributable cancers [17]. Consequently, universal vaccination and early detection remain cornerstone strategies for the prevention of the vast majority of HPV-related cancers.

#### Epstein-Barr Virus (EBV)

EBV is a ubiquitous HHV-4 primarily transmitted through saliva, although it can also spread via blood and organ transplants. Over 95% of healthy adults worldwide are infected with EBV, typically without overt symptoms; the majority of infections remain asymptomatic or result in benign, self-limiting disease [18]. However, EBV has been implicated in the pathogenesis of several malignancies, including but not limited to nasopharyngeal carcinoma, Burkitt lymphoma, Hodgkin lymphoma, various B- and T-cell lymphomas (including diffuse large B-cell lymphoma), and a subset of gastric carcinomas. A notably elevated incidence of nasopharyngeal carcinoma is observed in Southeast Asia, most prominently in southern China. This geographic predilection is thought to arise from an interplay among EBV infection, host genetic susceptibility, and environmental exposures, most notably the consumption of preserved foods [18].

#### Kaposi’s Sarcoma-associated Herpesvirus (KSHV)/Human Herpesvirus 8 (HHV-8)

KSHV, also known as HHV-8, is the primary pathogen responsible for KS, primarily transmitted through saliva, sexual contact, and blood [19]. KS is a rare but pathologically distinctive vascular endothelial cell tumor commonly observed in HIV-infected individuals or immunosuppressed patients following organ transplantation [20]. KSHV is also implicated in lymphoproliferative disorders such as primary exudative lymphoma (PEL) and multicentric Castleman disease (MCD) [19].

#### Hepatitis B Virus (HBV)

HBV infection remains one of the most important causes of hepatocellular carcinoma (HCC) worldwide and carries a particularly heavy burden in resource-limited regions of Asia and Africa, where 60%–80% of HCC cases may be associated with chronic HBV infection [21, 22]. HBV is transmitted primarily through mother-to-child transmission, blood exposure, and sexual contact.

In contrast to oncogenic viruses characterized by direct genomic integration, HBV is not known to encode classical oncogenes nor does it typically induce direct host genomic mutagenesis. Highly effective HBV vaccination programs have significantly reduced HBV infection rates and HBV-related HCC incidence in many regions worldwide [23]. The widespread adoption of HBV vaccination has reduced the prevalence of HBsAg (hepatitis B surface antigen) among children under 5 years old from over 5% to 0.9% [22, 24].

#### Hepatitis C Virus (HCV)

HCV infection is also a major global cause of HCC. HCV is primarily transmitted through blood. Similar to HBV, chronic HCV infection leads to persistent liver inflammation, fibrosis, and cirrhosis, significantly increasing the risk of HCC [25]. In recent years, the advent of direct-acting antiviral drugs (DAAs) has revolutionized the treatment landscape for HCV infection, markedly improving HCV clearance rates (cure rates). This development is anticipated to substantially diminish the future incidence of HCV-associated hepatocellular carcinoma (HCC) [26].

#### Merkel Cell Polyomavirus (MCPyV)

MCPyV is one of the most recently identified tumor viruses in human history, closely associated with Merkel cell carcinoma (MCC)—a rare yet highly aggressive neuroendocrine skin cancer [27, 28]. Approximately 80% of MCC cases are associated with MCPyV infection [27]. MCPyV infection is prevalent in the general population and typically asymptomatic; however, immunodeficiency (e.g., in the elderly, solid organ transplant recipients, or HIV-infected individuals) represents a key risk factor for MCC development [27].

#### Human T-lymphotropic Virus Type 1 (HTLV-1)

HTLV-1 was the first human retrovirus discovered and remains strongly associated with adult T-cell leukemia/lymphoma (ATLL) and HTLV-1-associated myelopathy/tropical spastic paraparesis (HAM/TSP) [29, 30]. Transmission occurs mainly through breastfeeding, blood contact, and sexual intercourse [30].

HTLV-1 is particularly informative as a model of long-latency viral carcinogenesis. It demonstrates that transformation may arise only after prolonged interaction among viral gene expression, host immune response, and chronic inflammatory signaling. The absence of a licensed vaccine also highlights the continuing importance of blood screening and prevention of mother-to-child transmission.

#### Human Immunodeficiency virus (HIV)

HIV is a retrovirus that targets CD4^+^ T lymphocytes, driving a progressive deterioration of immune competence that culminates in acquired immunodeficiency syndrome (AIDS). Transmission occurs primarily via sexual contact, exposure to infected blood or blood products (e.g., shared needles for intravenous drug use, unsafe injections), and mother-to-child transmission. Although HIV itself does not directly encode oncogenes or integrate into the host genome to induce carcinogenesis, the resulting immunosuppression substantially elevates the risk of developing various malignancies.

According to the World Health Organization (WHO) classification, AIDS-related tumors primarily fall into three categories: KS, non-Hodgkin lymphoma, and cervical malignancies. Among these, AIDS-related lymphoma is the most common AIDS-defining malignancy, accounting for 30–50% of HIV-associated cancers. The burden of malignancy extends beyond these defining conditions, with HIV-infected individuals demonstrating elevated incidence rates of anal cancer, Hodgkin lymphoma, and skin cancer, all of which exhibit higher incidence rates than in the general population [31]. As of 2021, approximately 40 million people worldwide were living with HIV, with sub-Saharan Africa bearing the heaviest burden, followed by Asia, Latin America, and the Caribbean [32]. Mortality rates for HIV-associated malignancies correlate closely with the stage at diagnosis, access to care, and the degree of immune reconstitution. Core strategies for mitigating the incidence and mortality of HIV-associated tumors include: ensuring universal access to antiretroviral therapy (ART) for immune reconstitution; promoting vaccination against oncogenic co-infections such as HPV and KSHV; and intensifying tumor screening efforts in this population, including cervical cytology and anoscopy [32]. (Table 1).

**Table 1:** Transmission modes, epidemiological features, and prevention/control information of major oncogenic viruses

Virus	Transmission Modes	Global Epidemiological Features / Geographic Distribution	Typically Associated Cancers	Antigen Target	Immune Mechanism	Vaccine Development Status	Core Antiviral Drugs	Ref
HPV	Primarily sexual contact; minor indirect contact	High prevalence globally. Leading regional shares: Asia (54%), Africa (19%), Americas (16%)	Cervical cancer, vulvar cancer, anal cancer, oropharyngeal cancer	L1 VLP, E6/E7	Neutralizing antibodies; CD8⁺ CTL	Licensed (Bivalent / Quadrivalent/Nonavalent)	No specific antiviral drugs; prevention is key	[14–17, 33–35]
EBV	Primarily via saliva; minor via blood/organ transplant	> 95% of adults worldwide infected. Nasopharyngeal carcinoma high in Southeast Asia & Southern China	Nasopharyngeal carcinoma, Burkitt lymphoma, Hodgkin lymphoma, gastric cancer	gp350, gH/gL/Gb, gp42	CD8⁺ CTL; multi-antigen humoral immunity	Preclinical / early clinical	No specific drugs; symptomatic treatment	[21–24, 36]
KSHV	Saliva, sexual contact, blood	Low prevalence globally; high in HIV + and immunocompromised populations	Kaposi's sarcoma, primary effusion lymphoma, multicentric Castleman disease	LANA, glycoproteins, multi-antigen	Latent + lytic antigen CTL activation	Preclinical	No specific drugs; immune reconstitution aids control	[19, 20, 37, 38]
HBV	Mother-to-child, blood exposure, sexual contact	High in less developed regions of Asia & Africa. Global HBsAg prevalence in children < 5 yrs dropped to 0.9%	Hepatocellular carcinoma	HBsAg, HBcAg	Neutralizing antibodies; T-cell immunity	Approved + clinical trials	Nucleos(t)ide analogs (e.g., Entecavir, Tenofovir)	[21–24, 39–41]
HCV	Primarily blood (shared needles, unsafe injections)	Widespread globally; no distinct geographic clustering	Hepatocellular carcinoma	Core, NS3, NS5A	CD8⁺ CTL; reduced Treg	Preclinical	Direct-acting antivirals (DAAs) (e.g., Sofosbuvir, Velpatasvir)	[25, 26, 31, 42–44]
MCPyV	Route unknown; presumed skin contact	High seroprevalence in general population; ubiquitous worldwide	Merkel cell carcinoma	T-Ag, small T antigen	CTL targeting T-antigen	Preclinical	No specific drugs; surgical excision primary	[27, 28, 45]
HTLV-1	Breastfeeding, blood contact, sexual contact	High in southwestern Japan, Caribbean, parts of Africa	Adult T-cell leukemia/lymphoma, HAM/TSP	Tax, HBZ, gp21, gp46, gag	Mucosal + systemic T-cell responses	Preclinical	No specific drugs; chemotherapy primary (limited efficacy)	[29, 30, 46]
HIV	Sexual contact, blood, mother-to-child	Heaviest burden in sub-Saharan Africa (~ 70% global), then Asia, Latin America	Kaposi's sarcoma, non-Hodgkin lymphoma, cervical cancer	Env (gp160), V3 loop, bnAb epitopes	Broad neutralizing antibodies (bnAbs)	Clinical trials	Antiretroviral drugs (e.g., Raltegravir, Emtricitabine/Tenofovir)	[31, 32, 47–49]

VLP, Virus-Like Particle; CTL, Cytotoxic T Lymphocyte; CD8⁺, Cluster of Differentiation 8 Positive; Treg, Regulatory T Cell; HBsAg, Hepatitis B Surface Antigen; HBcAg, Hepatitis B Core Antigen; bnAbs, Broadly Neutralizing Antibodies; Env, Envelope protein; gH/gL/gB, gp42, gp350, Designations for specific viral glycoproteins; LANA, Latency-Associated Nuclear Antigen; T-Ag, Large T antigen; Tax, HBZ, Designations for viral regulatory proteins; NS3, NS5A, Nonstructural Protein 3/5A; HAM/TSP, HTLV-1-Associated Myelopathy / Tropical Spastic Paraparesis; DAAs, Direct-Acting Antivirals

### Emerging viral threats and cancer risk

While the role of classic tumor viruses in cancer etiology has been well established, newly emerging and re-emerging viral pathogens persist as a threat to global public health, and their potential long-term carcinogenic risks have drawn considerable scrutiny. Among these, SARS-CoV-2 (the novel coronavirus) has attracted particular interest, not only due to its role in triggering the global COVID-19 pandemic but also for its emerging implications for chronic disease sequelae. Although SARS-CoV-2 is not a direct carcinogenic virus, multiple epidemiological studies have suggested that the chronic inflammatory state and immune dysfunction following infection may be associated with elevated risks of lung cancer [50, 51], skin cancer [52], and hematological malignancies [53]. Notably, these associations display marked geographic heterogeneity and distinct risk-stratification patterns.

Other emerging viruses raise similar but even more preliminary questions. Human polyomaviruses such as HPyV6, HPyV7, JCPyV, and BKPyV are highly prevalent in the general population, and some may reactivate in immunocompromised individuals [54, 55]. Associations with tumors have been reported for JCPyV and BKPyV, but the mechanistic and causal evidence remains incomplete [56, 57]. Respiratory syncytial virus (RSV) is another example: although primarily recognized as a respiratory pathogen, it poses substantial risk in patients with hematologic malignancies and may contribute indirectly to adverse oncologic outcomes [58, 59]. Highly pathogenic avian influenza viruses, monkeypox virus, and chikungunya virus have also been discussed as potential contributors to tumor-promoting conditions through immune activation, cytokine storms, tissue repair, or chronic inflammatory sequelae, but direct evidence of carcinogenicity remains insufficient [60–63].

The broader lesson is that the boundary between “oncogenic virus” and “non-oncogenic virus with potential long-term oncologic relevance” may not always be absolute. For newly emerging pathogens, the key scientific question is often not whether they act as classical transforming viruses, but whether they create persistent biological conditions—such as chronic inflammation, fibrosis, immune dysfunction, or impaired tissue repair—that increase vulnerability to malignant evolution. Therefore, sustained epidemiologic surveillance, mechanistic studies, and long-term follow-up cohorts are essential before firm conclusions can be drawn.

## Mechanisms and consequences of virus-induced cancer

**Fig. 2:**
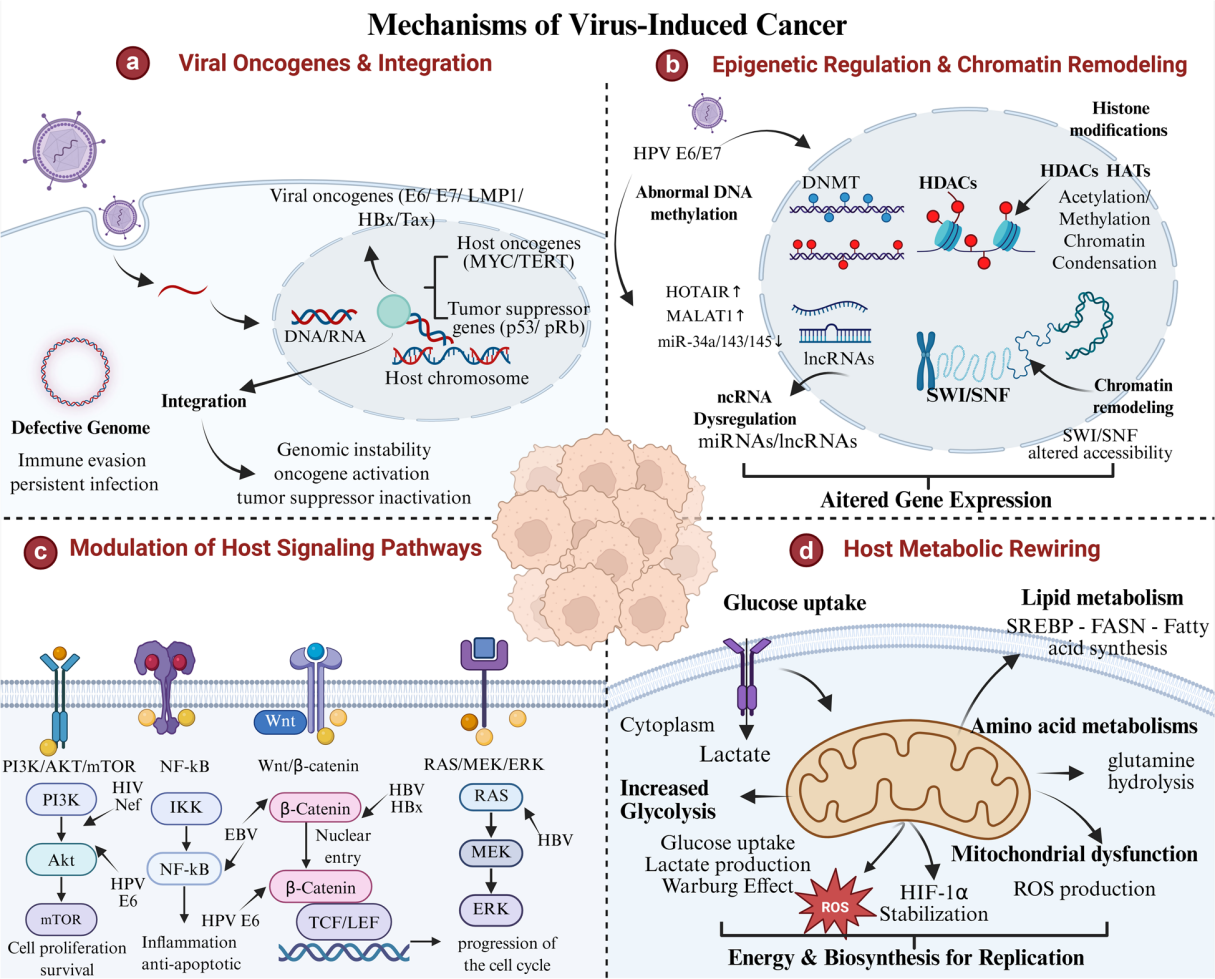
Mechanisms of virus-induced cancer. Viral infection induces cancer through four primary pathways. **a** Viral Oncogenes & Integration: Viral genetic material enters host cells and may integrate into host chromosomes. Expression of viral oncogenes (e.g., HPV E6/E7, EBV LMP1, HBV HBx, HTLV-1 Tax) activates host oncogenes (e.g., MYC, TERT) and inactivates tumor suppressor genes (e.g., p53, pRb). Most viruses exhibit characteristic genetic defects during tumorigenesis, which collectively contribute to genomic instability, immune evasion, and persistent infection. **b** Epigenetic Regulation & Chromatin Remodeling: Oncogenic viruses frequently disrupt host epigenetic regulatory networks to facilitate their own latency and replication while promoting cellular malignant transformation. Viral proteins modulate the epigenetic machinery, including DNA methyltransferases (DNMTs) and histone deacetylases (HDACs), causing abnormal DNA methylation and histone modifications. This is accompanied by chromatin remodeling (e.g., via SWI/SNF complexes) and dysregulation of non-coding RNAs (ncRNAs), such as upregulation of long non-coding RNAs (HOTAIR, MALAT1) and downregulation of tumor-suppressing miRNAs, ultimately leading to altered gene expression. **c** Modulation of Host Signaling Pathways: To survive and replicate within host cells, viruses often hijack key intracellular signaling cascades—Including PI3K/AKT/mTOR, NF-κB, Wnt/β-catenin, and RAS/MEK/ERK pathways—to promote cell proliferation, survival, inflammation, and anti-apoptotic responses. **d** Host Metabolic Rewiring: Viral infection induces metabolic restructuring in host cells to support viral replication and meet the high metabolic demands of transformed cells. This primarily occurs through multiple metabolic pathways, including glycolysis, lipid metabolism, amino acid metabolism, folate metabolism, and lactate metabolism, thereby contributing to carcinogenesis. The Warburg effect and other metabolic pathways provide essential energy and biosynthetic precursors for viral replication and tumor growth

Viral infections are central to the initiation and progression of multiple human cancers. Virus-induced tumorigenesis is not driven by a single molecular event, but rather by a multilayered and reciprocal process in which direct viral actions interact with host genomic, epigenetic, metabolic, and microenvironmental responses. In broad terms, these mechanisms include direct pathways—such as viral genome integration, sustained oncogene expression, and induction of genomic instability—as well as indirect pathways involving epigenetic remodeling, aberrant activation of host signaling cascades, metabolic rewiring, and progressive reshaping of tissue homeostasis. Together, these virus-induced alterations endow infected cells with hallmark malignant traits, including sustained proliferative signaling, resistance to apoptosis, enhanced plasticity, and long-term survival (Fig. 2).

### Viral oncogenes and host genome integration

Viral oncogene expression and the integration of viral genomes are well-established drivers of tumorigenesis. Multiple oncogenic viruses sustain aberrant oncogene expression through integration into the host genome or the establishment of long-term cellular latency, thereby perturbing chromatin architecture and genomic stability. This persistent interference disrupts core regulatory networks in host cells, ultimately inducing genomic instability, uncontrolled cell cycle progression, and malignant transformation [64–66]. Based on the primary mode of viral carcinogenesis, virus-mediated oncogenic mechanisms can be summarized into three fundamental patterns: integration-mediated carcinogenesis centered on genomic integration; latent/epigenetic regulation-mediated mechanisms characterized by latent infection and chromatin regulation; and immunodeficiency-cooperative mechanisms primarily driven by immune deficiency.

#### Integrated Carcinogenic Mechanism

In integration-mediated carcinogenesis, stable incorporation of the viral genome into the host chromosome represents a critical initiating event. The integration process itself can induce insertion mutations and chromosomal structural abnormalities, often accompanied by disruption of viral regulatory genes, leading to uncontrolled expression of viral oncogenes. Persistently expressed viral oncoproteins further disrupt host cell cycle, DNA repair, and signaling pathways, ultimately causing systemic imbalance in the host regulatory network and driving malignant transformation and tumor progression [64, 67]. Thus, integration acts not only as a structural genomic insult, but also as a mechanism for establishing sustained oncogenic signaling.

During early HPV infection, the viral genome typically exists as free circular DNA within the host epithelial cell nucleus. In late stages of persistent infection, HPV DNA often integrates into the host genome [67]. This integration event is considered a key molecular node in the development of cervical cancer and certain head and neck squamous cell carcinomas. HPV DNA integration is frequently accompanied by fragmentation or deletion of the viral E2 gene, whose protein normally suppresses transcription of the oncogenes E6 and E7 [65]. Loss of E2 function leads to sustained high expression of E6/E7: E6 promotes degradation of the tumor suppressor protein p53, weakening DNA damage responses and apoptosis; E7 binds and inactivates pRb, releasing its inhibition of E2F and causing abnormal entry into the S phase [64, 65]. These changes collectively disrupt cell cycle checkpoints, induce genomic instability, and lay the groundwork for cellular immortalization and malignant transformation. Furthermore, integration events can activate neighboring host proto-oncogenes (e.g., MYC, CCND1), further enhancing proliferative advantage [68, 69].

HBV-associated hepatocarcinogenesis reflects a related but not identical pattern. HBV DNA can integrate randomly into host chromosomes, and some insertion sites occur near oncogenes or regulatory regions [70]. Unlike HPV, HBV integration is not obligatory for tumorigenesis; however, its high frequency during chronic infection provides a persistent basis for disruption of hepatocyte genomic architecture and transcriptional regulation. When integration occurs, the viral X gene is frequently retained, resulting in sustained HBx expression. HBx activates proliferative signaling pathways such as NF-κB, AP-1, and AP-2, suppresses apoptotic pathways including TNF-α- and Fas-mediated cell death, and perturbs the balance between tumor suppressors and oncogenes [70, 71]. Particularly important is HBV insertion into the TERT promoter, which directly enhances TERT expression and contributes to replicative immortality [70–72]. Thus, in HBV-associated tumors, genomic instability and persistent viral oncogenic signaling cooperate to drive hepatocellular carcinoma.

In addition to HPV and HBV, other oncoviruses promote malignant transformation through stable genomic integration and sustained expression of key oncoproteins. For HTLV-1, genomic integration constitutes an essential step within the replication cycle. The integrated virus persistently expresses Tax and HBZ in infected T cells, which synergistically activate pro-proliferative signals like NF-κB, disrupt the cell cycle, and suppress apoptosis, thereby driving the development of adult T-cell leukemia/lymphoma [73–76]. Similarly, in MCC, the MCPyV genome integrates into the host genome in a monoclonal form, accompanied by a C-terminal truncation mutation in the large T antigen (LT). Although this truncation ablates viral replication competence, it spares the pRb‑binding motif of LT. This retained domain persistently interferes with host cell-cycle regulation to drive aberrant proliferation, thereby contributing to tumorigenesis [77].

#### Latent/chromatin regulation-mediated carcinogenesis mechanism

Unlike integration-dependent viruses, latent/chromatin-regulated viruses do not rely on extensive genomic integration. Instead, they establish a long-term latent infection state within host cells, stably maintaining their genome as free episomes within the nucleus [78]. Through a restricted latent gene expression program, they continuously interfere with the host cell's signaling and transcriptional regulatory networks, thereby creating favorable conditions for the formation and maintenance of a tumorigenic phenotype.

EBV serves as a canonical example of latent-regulatory carcinogenesis. Within host cells, EBV establishes a predominantly latent infection, with the viral genome persisting as a nuclear episome. Long-term immune evasion is achieved through the temporally regulated expression of latent-associated genes [79, 80]. In B cells, the EBV-encoded latent membrane protein 1 (LMP1) mimics a persistently activated CD40 receptor, thereby continuously activating pro-survival and pro-proliferative signaling pathways such as NF-κB. Concurrently, LMP2A disrupts B-cell receptor signal transduction, thereby allowing infected cells to retain survival and proliferative capacity in the absence of cognate antigen stimulation [79]. Furthermore, EBV finely regulates viral and host gene expression through non-coding RNAs such as the BART microRNA (miRNA) cluster, suppressing lytic gene expression and sustaining latency to achieve long-term remodeling of the host transcriptional program [81]. Within this framework of latency and transcriptional regulation, EBV-infected cells progressively acquire immortalized characteristics, evade immune surveillance, and develop into various malignancies—including Burkitt lymphoma, diffuse large B-cell lymphoma, extranodal NK/T-cell lymphoma, nasopharyngeal carcinoma, and gastric carcinoma—within specific tissue microenvironments [80–82].

Similarly, during latency, KSHV primarily relies on the latent-associated nuclear antigen (LANA) to maintain the viral genome and regulate host cell functions. Studies indicate that LANA directly binds to the tumor suppressor protein p53 and inhibits its transcriptional activity, thereby blocking p53-mediated apoptosis. This helps infected cells evade immune clearance and gain a sustained survival advantage [83]. Furthermore, KSHV suppresses antigen presentation and modulates chemokine signaling through the expression of latency-associated proteins such as K5 and K6, thereby shaping an immunosuppressive tumor microenvironment that facilitates the long-term survival and clonal expansion of infected cells [78]. This regulatory pattern, characterized by sustained expression of latent genes, enables KSHV to promote the maintenance and expansion of pro-tumor phenotypes by stably interfering with key host regulatory pathways, independent of extensive genomic integration [84].

#### Immunodeficiency-synergistic carcinogenesis mechanism

The core mechanism of immunodeficiency-associated carcinogenesis does not reside in the direct transformation of host cells by the virus; rather, it stems from the weakening of immune surveillance, which in turn fosters the amplification of latent oncogenic viruses or malignant clones.

A hallmark feature of HIV infection is the persistent decline in CD4⁺ T-lymphocyte counts, which ultimately culminates in acquired immunodeficiency syndrome (AIDS) and sustained immune dysfunction [85]. In this context, latent oncogenic viruses harbored by the host—including EBV, KSHV, and HPV—exhibit heightened susceptibility to reactivation or persistent infection. This facilitates the acquisition of proliferative advantage and evasion of apoptosis by associated cellular clones [86–88]. This immunosuppressive effect not only amplifies the oncogenic potential of latent viruses but also provides a favorable environment for malignant clones to accumulate genetic or epigenetic mutations, thereby indirectly promoting tumorigenesis. Clinical studies demonstrate significantly elevated incidence rates of KS, non-Hodgkin lymphoma, and cervical cancer among HIV-infected individuals, fully illustrating the clinical significance of immune deficiency synergizing carcinogenesis [87–89]. Thus, in virus-associated tumorigenesis, HIV functions more as a “promoter” and “amplifier.” By weakening the host immune system, it indirectly assists latent viruses or malignant clones in stably acquiring oncogenic phenotypes, thereby supporting tumor initiation and progression.

Similarly, while HCV does not cause systemic immunodeficiency, its chronic infection leads to persistent imbalance in the hepatic immune microenvironment [90]. HCV enables the long-term survival and accumulation of genetic and epigenetic abnormalities in precancerous cells by inducing T-cell functional exhaustion, suppressing NK cell antitumor activity, and weakening immune surveillance of abnormal hepatocytes. Concurrently, chronic inflammation and immune evasion synergistically promote the initiation and progression of hepatocellular carcinoma [91]. Therefore, in both HIV- and HCV-associated carcinogenesis, the key principle is that impaired immune surveillance enables infected or abnormal cells to remain within the tissue long enough to evolve toward malignancy.

In summary, viral oncogene expression and viral genome integration constitute the core initiating mechanisms of virus-induced tumorigenesis. Integrating viruses directly drive malignant transformation by disrupting host genomic integrity and activating viral oncogenes; Latent viruses rely on long-term stable gene expression programs to continuously remodel host transcription and chromatin states; whereas immunodeficiency-synergistic viruses indirectly promote the development of various virus-associated tumors by weakening immune surveillance. These fundamental mechanisms establish a crucial molecular foundation for subsequent discussions on expanded mechanisms such as epigenetic regulation, metabolic reprogramming, and tumor microenvironment remodeling in later chapters.

### Epigenetic regulation and chromatin remodeling

Epigenetic remodeling is a central mechanism by which oncogenic viruses stabilize persistence and promote malignant transformation. Unlike irreversible genetic mutations, epigenetic alterations provide viruses with a flexible means of reshaping host-cell behavior, suppressing defense programs, and sustaining oncogenic phenotypes without necessarily altering DNA sequence. Although different viruses use distinct viral proteins and host cofactors, they converge on a common strategy: restructuring the transcriptional and chromatin landscape of infected cells so that survival, persistence, and malignant evolution become mutually reinforcing.

#### Silent tumor suppressor gene

Silencing of host tumor suppressor genes via epigenetic mechanisms is directly induced by multiple oncogenic viruses, a process that contributes to tumorigenesis. In this context, DNA methylation, histone modification, and non-coding RNA dysregulation act together to suppress transcription of genes that would otherwise limit proliferation, induce apoptosis, or preserve genomic integrity.

In HPV-associated tumors, the virally encoded E6 and E7 oncoproteins not only disrupt the p53 and pRb signaling but also promote promoter hypermethylation and consequent silencing of tumor suppressor genes—such as APC1 and CDH1—through the upregulation of DNA methyltransferase 1 (DNMT1) activity. Concurrently, E7 recruits histone deacetylases and associated complexes, thereby altering histone modification landscapes and promoting chromatin compaction. These modifications collectively reduce the transcriptional activity of tumor suppressor genes [92–95]. Additionally, HPV infection can induce dysregulation of non-coding RNA expression within host cells. HPV upregulates the expression of oncogenic long non-coding RNAs (lncRNAs) such as HOX antisense regulatory RNA (HOTAIR) and metathrombin-associated long non-coding RNA 1 (MALAT1), while downregulating the expression of tumor suppressor microRNAs (miRNAs) including microRNA-34a (miR-34a), microRNA-143 (miR-143), and microRNA-145 (miR-145), thereby promoting cancer progression [96, 97]. Such epigenetic perturbations not only enforce the silencing of key tumor suppressor genes but also establish a cellular milieu permissive to sustained viral replication and host cell malignant transformation.

Similarly, EBV upregulates DNMT1 and DNMT3A/3B expression in host cells via its latent membrane proteins LMP1, LMP2A, and nuclear antigen EBNA3 family proteins, promoting CpG island methylation through signaling pathways such as STAT3 and JNK-AP1. This mechanism induces hypermethylation and silencing of multiple tumor suppressor gene promoters (e.g., CDH1, PTEN). Concurrently, EBV proteins recruit histone modification complexes like PRC2, increasing inhibitory histone marks such as H3K27me3 to further suppress tumor suppressor gene transcription [98, 99]. These epigenetic alterations manifest as the CpG island methylation phenotype (CIMP) in EBV-associated nasopharyngeal carcinoma and EBV-associated gastric carcinoma, significantly promoting malignant transformation of host cells.

HBV and HCV show that this strategy is not restricted to DNA tumor viruses. HBV's HBx protein indirectly upregulates DNMT expression by activating signaling pathways like NF-κB and STAT3, altering host genomic methylation patterns and thereby affecting tumor suppressor gene transcription [100–102]. The HCV core protein also upregulates DNMT1 and DNMT3B activity, promoting abnormal methylation-mediated silencing of host tumor suppressor genes, though the precise mechanisms require further validation [103, 104]. These lentiviruses and hepatitis viruses achieve tumor suppressor gene silencing by regulating host DNA methylation and histone modifications, thereby establishing a foundation for sustained viral infection, immune evasion, and cellular malignant transformation.

#### Suppression of immune-related genes

A second major consequence of viral epigenetic remodeling is suppression of host immune-related genes. Viruses suppress host immune genes through epigenetic regulation to achieve immune evasion, thereby maintaining long-term latency or persistent infection.

KSHV illustrates this mechanism well. KSHV-encoded SOX proteins inhibit AIM2 inflammasome activation, while its latency-associated nuclear antigen (LANA) recruits histone modification complexes to silence host immune-related genes (e.g., IL-1β, IFN-β), thereby weakening the host's early innate immune response [105–107]. KSHV also upregulates immunosuppressive signaling molecules (e.g., PD-L1) via vIL-6 and other viral factors, enabling infected cells to evade T/NK cell recognition and thereby sustain latent infection and malignant transformation [108].

During HIV infection, the Tat protein interferes with DNA repair and transcriptional regulatory proteins like Tip60, reducing expression of host immune-related genes (e.g., IFN-β, CD4). Concurrently, the Nef protein upregulates PD-L1 and activates the MEK/ERK signaling pathway to suppress T/NK cell activity, enabling infected cells to evade immunity and promoting hematopoietic malignancies [86–88].

Beyond silencing tumor suppressor pathways, HPV, HBV, HCV, and EBV also achieve immune evasion by suppressing host immune-related genes, a process that facilitates tumorigenesis. The E5 protein of HPV reduces cell-surface expression of MHC-I molecules, whereas the E6/E7 oncoproteins indirectly suppress IFN-κ and other immune-related genes through the regulation of DNMTs and histone-modifying enzymes [66, 92, 94]. HBV's HBx protein upregulates the immune checkpoint ligand CD155 and disrupts antigen presentation-related genes, thereby weakening the immune clearance capacity of NK and T cells [100, 101]. HCV promotes the long-term survival and accumulation of genetic and epigenetic abnormalities in precancerous cells through the induction of T-cell exhaustion, the suppression of NK cell activity, and the attenuation of immune surveillance targeting abnormal hepatocytes [90, 109]. EBV's LMP1, LMP2A, and EBNA3 family proteins of EBV can upregulate PD-L1 and IL-10 while silencing proinflammatory cytokine genes (e.g., CXCL10, IFN-γ), further suppressing T cell function [79, 98, 99].

Collectively, these four viruses establish a synergistic intracellular environment conducive to tumorigenesis, achieved through the dual actions of silencing tumor suppressor genes and attenuating immune surveillance.

#### Shaping cell fate

Beyond the silencing of tumor suppressor and immune-related genes, host cell fate may be reshaped by viral mechanisms that promote stemness, epithelial-mesenchymal transition (EMT), and aberrant proliferation—processes that collectively drive tumorigenesis and disease progression.

For instance, the E6 and E7 oncoproteins of HPV modulate the epigenetic landscape of cell cycle genes and tumor suppressor promoters. They exert these effects by liberating E2F transcription factor activity and recruiting HDAC and HAT complexes, thereby driving persistent proliferation and apoptotic evasion in infected cells [92, 94, 95, 110]. HBV's HBx protein regulates stem cell-associated genes (e.g., SNAI2) and the NF-κB pathway, sustaining hepatocellular carcinoma stem cell properties and enhancing cellular survival [100, 101]. EBV employs latent proteins—including LMP1 and the EBNA3 family—to orchestrate chromatin states and epigenetic modifications. This establishes a chromatin milieu permissive for viral latency and sustained host cell proliferation while simultaneously blocking apoptotic signals [98, 99, 106, 107]. In addition, the LANA protein of KSHV recruits host epigenetic modifying complexes to establish and preserve chromatin architecture. This action suppresses a subset of host gene expression, thereby ensuring viral latency while endowing infected cells with both anti-apoptotic traits and a sustained proliferative capacity [106, 107].

Through these mechanisms, the virus not only creates a favorable environment for its own latency or replication but also reprograms host cells into a pro-cancerous state. This includes enhancing stem-like characteristics, promoting epithelial-mesenchymal transition (EMT), and driving abnormal proliferation, thereby establishing sustainable oncogenic potential.

### Modulation of host signaling pathways

Oncogenic viruses often hijack host cell signaling pathways to promote their own survival, replication, and latency, while concurrently propelling host cells toward malignant conversion. The abnormal activation or inhibition of these signaling pathways constitutes the core mechanism by which viruses induce cellular proliferation, anti-apoptosis, migration, and stemness maintenance. Overall, viruses primarily exert their effects through three common signaling pathway frameworks: the PI3K/AKT/mTOR and RAS/RAF/MEK/ERK pathways promote proliferation, survival, metabolic reprogramming, and angiogenesis; the NF-κB/STAT3 pathway mediates chronic inflammation, suppresses immune responses, and shapes the pro-cancer microenvironment; while the Wnt/TGF-β pathway maintains stemness phenotypes, drives EMT, and regulates cellular plasticity. Viruses access these common pathways through diverse strategies to achieve extensive regulation of host cells.

The PI3K/AKT/mTOR and RAS/RAF/MEK/ERK signaling pathways function as central hubs in orchestrating virus-driven proliferation and metabolic reprogramming. HPV's E6 protein directly activates PI3K while suppressing expression of its negative regulator, phosphatase and tensin homolog (PTEN), thereby promoting AKT phosphorylation and activating downstream mTOR signaling to accelerate protein synthesis and cell growth [103]. EBV's LMP1 suppresses PTEN expression via miR-21, while LMP2A promotes angiogenesis through the PI3K/AKT/mTOR/hypoxia-inducible factor 1α (HIF-1α) signaling axis [109]. HBV's HBx protein promotes cell proliferation via mTOR signaling and the RAS/RAF/MEK/ERK pathway [111, 112], while HCV's core and NS5A proteins similarly activate the PI3K/AKT/mTOR pathway, inducing hepatocyte proliferation and metabolic reprogramming [113]. KSHV engages the AKT/mTORC1 signaling axis through its G protein-coupled receptor, thereby driving angiogenesis and metabolic reprogramming [114]. Likewise, the Tat and Nef proteins of HIV enhance angiogenesis, cell proliferation, and metabolic activity, while simultaneously suppressing apoptosis, through activation of the PI3K/AKT/mTOR signaling pathway [113, 115, 116]. The common principle is that viruses repeatedly access these pathways to create a metabolic and proliferative state favorable to both persistence and transformation.

In the context of the NF-κB/STAT3 signaling pathway, viruses foster a pro-tumorigenic microenvironment by driving chronic inflammation and attenuating immune surveillance. EBV's LMP1 protein mimics tumor necrosis factor receptor signaling, recruits TRAF proteins, activates the IKK complex, and drives NF-κB translocation to the nucleus. These events upregulate anti-apoptotic genes including Bcl-2 and inhibitors of apoptosis proteins (IAPs); concurrently, they suppress miR-203 expression, promote EMT, and activate JAK/STAT signaling, thereby establishing a self-perpetuating pro-proliferative loop [85, 109]. HCV infection elicits production of cytokines including TNF-α and IL-6, which subsequently activate NF-κB, perpetuating a chronic inflammatory milieu and enhancing cell survival [117]. Similarly, SARS-CoV-2 infection triggers an excessive release of proinflammatory mediators such as IL-6 and TNF-α, which activates NF-κB/STAT3 signaling, culminating in a cytokine storm and impairment of antitumor immune defenses [118, 119]. These observations suggest that inflammation-associated signaling is not only a consequence of viral infection, but also a mechanistic amplifier of tumor-promoting conditions.

In the Wnt/β-catenin and TGF-β signaling pathways, viruses alter cellular plasticity by maintaining stem-like phenotypes and driving EMT. HPV E6 stabilizes β-catenin and activates Wnt signaling, while E5 suppresses extrinsic apoptosis and promotes EMT [103]. EBV-encoded products such as LMP1 and LMP2A cause β-catenin accumulation, nuclear translocation, and downstream gene expression alterations, participating in tumor-related processes including EMT and cell proliferation [109]. HBV HBx activates Wnt/β-catenin signaling by suppressing miR-26a, maintaining a liver cancer stem-like phenotype and EMT [70]. EBV's miR-BART7-3p indirectly activates β-catenin by targeting PTEN, accelerating EMT progression [109]. In this context, these pathways serve as key mechanisms through which viruses shift infected cells from controlled tissue programs toward invasive, adaptable malignant states.

In addition to the aforementioned signaling pathways, the cGAS–STING innate immune pathway and the AMPK metabolic regulation pathway are also implicated. Specifically, the cGAS–STING pathway is associated with virus-induced upregulation of SMPDL3B expression, thereby suppressing the host innate immune response [120]. Regarding the AMPK pathway, HCV can influence the LKB1–AMPK–TSC1/2 axis, suggesting its potential role in virus-mediated metabolic reprogramming [121].

In summary, oncogenic viruses hijack multiple common signaling pathways to achieve core pro-cancer phenotypes such as cellular proliferation, anti-apoptosis, stemness maintenance, EMT, and immune evasion. Although viral strategies differ, they operate within a common framework, endowing host cells with long-term carcinogenic potential. Future research integrating multi-omics data and emerging technologies may deepen our understanding of virus-host signaling interactions, offering potential strategies for developing targeted therapies (Table 2).

**Table 2:** Therapeutic strategies targeting viral and host oncogenic pathways for virus-associated tumors

Virus	Key Viral Oncogenic Factors	Virus-Targeting Strategies	Key Host Oncogenic Pathways	Host-Targeting Strategies	Ref
HPV	E6/E7	CRISPR/Cas9, TALEN, ZFN for targeted cleavage of integrated viral sequences	p53/pRb, PI3K-AKT-mTOR, Wnt/β-catenin, DNA damage response	PARP inhibitors (e.g., veliparib), in combination with cisplatin	[94, 95, 122, 123]
EBV	LMP1, LMP2A, EBNA1, EBNA3, BART miRNAs	CRISPR/Cas9 for site-specific cleavage of EBV cccDNA	NF-κB, JAK/STAT, PI3K-AKT, PD-L1	NF-κB/JAK/STAT pathway inhibitors (synergistic with antiviral therapies)	[98, 109, 124, 125]
HBV	HBx protein, PreS/S mutant	CRISPR/Cas9, TALEN for cccDNA degradation	NF-κB, STAT3, Wnt/β-catenin, TERT	Pegylated interferon, IFN-α/TLR agonists (e.g., GS-9688)	[70–72, 100, 101, 126, 127]
HCV	Core protein, NS3/NS4A protease	Direct-acting antiviral agents (DAAs) to clear HCV	PI3K-AKT-mTOR, HIF-1α, AMPK	Telomerase/Wnt/β-catenin pathway inhibitors (combined with DAAs)	[103, 104, 109, 128–131]
HTLV-1	Tax, HBZ proteins, HTLV-1 mRNA	Antisense therapy targeting HTLV-1 mRNA; immunotherapy targeting Tax protein	NF-κB, p53 inhibitor	Proteasome inhibitors (bortezomib), NF-κB pathway inhibitors	[74, 75, 132]
KSHV	LANA, vGPCR, K5/K6	Target LANA to disrupt viral episome maintenance; vGPCR antagonists	mTOR, HIF-1α	mTOR inhibitors (rapamycin)	[78, 108, 123, 133–135]
MCPyV	LT-ag, sT-ag	CRISPR/Cas9 for targeted disruption of integrated viral T-antigen genes	pRb-E2F, Glycolysis-lactate pathway	Immune checkpoint inhibitor (PD-1/PD-L1)	[136–140]
HIV	Tat, Nef, Vpu	Highly Active Antiretroviral Therapy (HAART) to control HIV viral load	PI3K-AKT-mTOR, Immune checkpoint	Immune reconstitution, Immune checkpoint inhibitors	[113, 115, 116, 141, 142]

CRISPR, clustered regularly interspaced short palindromic repeats; TALEN, transcription activator-like effector nuclease; ZFN, zinc finger nuclease; p53, tumor protein p53; pRb, retinoblastoma protein; PARP, poly (ADP-ribose) polymerase; LMP1, latent membrane protein 1; LMP2A, latent membrane protein 2 A; EBNA1/3, Epstein-Barr virus nuclear antigen 1/3; BART, BamHI A Rightward Transcripts; JAK, janus kinase; STAT, signal transducer and activator of transcription; RIG-I, retinoic acid-inducible gene I; TLR, toll-like receptor; vGPCR, viral G protein-coupled receptor; mTOR, mechanistic target of rapamycin; STING, stimulator of interferon genes;PD-1, programmed death-1; NF-κB, nuclear factor-κB; HIF-1α, hypoxia-inducible factor 1 alpha; Tat, trans-activator of transcription; Nef, negative regulatory factor; Vpu, viral protein U

### Host metabolic rewiring under viral infection

Virus-mediated metabolic reprogramming generally stems from the aberrant activation of signaling pathways. For instance, the PI3K/AKT, MAPK, NF-κB, and Wnt signaling axes drive key transcriptional and enzymatic programs underlying metabolic pathways, the resulting metabolic alterations then manifest as disruptions in energy balance, biosynthesis, and redox homeostasis. Viral infection can systematically remodel host cell metabolic pathways, supplying energy and biosynthetic substrates for viral replication and sustained proliferation of transformed cells, thereby fueling tumorigenesis and progression [143].

**Fig. 3:**
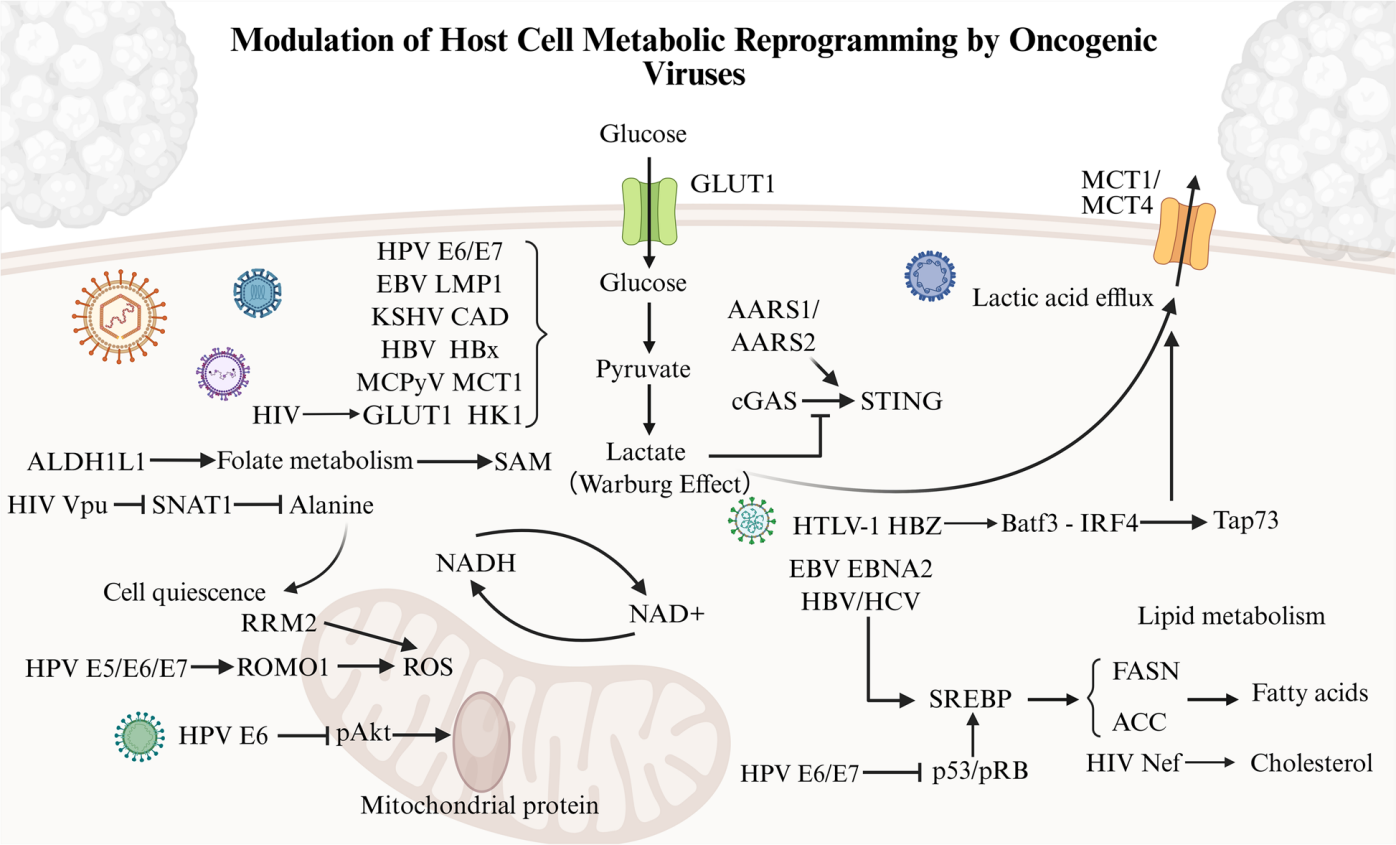
Modulation of host cell metabolic reprogramming by oncogenic viruses. Oncogenic viruses can reshape host cell metabolism to sustain their own replication and promote cancerous transformation. At the level of glucose metabolism, viral effector molecules such as HPV E6/E7 and EBV LMP1 regulate glucose uptake pathways by modulating glucose transporters GLUT1 and hexokinase 1 (HK1), and activate the Warburg effect to convert pyruvate from glycolysis into lactate. MCPyV-encoded ST and HTLV-1's HBZ RNA can upregulate MCT1/4 to promote lactate efflux, preventing intracellular toxic accumulation and indirectly facilitating cancer cell development. Furthermore, lactate can inhibit the cGAS-STING signaling axis via the AARS1/AARS2 pathway, concurrently weakening the host's innate antiviral immunity. Simultaneously, metabolic pathways such as folate metabolism, lipid metabolism, and mitochondrial function are all influenced by various viral factors. In amino acid and nucleotide metabolism, HIV Vpu downregulates SNAT1 to induce cellular quiescence, while ALDH1L1 mediates the link between folate metabolism and SAM synthesis. Regarding mitochondrial homeostasis, HPV E5/E6/E7 upregulate ROMO1 to elevate ROS levels, and HPV E6 inhibits pAkt activity, collectively disrupting mitochondrial function. In lipid metabolism pathways, EBV EBNA2 activates SREBP to drive FASN/ACC-dependent fatty acid synthesis, while HIV Nef participates in cholesterol homeostasis regulation. Additionally, HTLV-1 HBZ targets Tap73 via the Batf3-IRF4 axis, while HPV E6/E7 inactivates tumor suppressor proteins p53/pRB. These synergistic regulatory networks provide critical support for virus-mediated metabolic reprogramming of host cells

Unlike aberrations restricted to single metabolic pathway abnormalities, virus-mediated metabolic reprogramming operates as a highly coordinated network and manifests primarily through three core pathways: enhanced glycolysis, reshaped lipid and cholesterol metabolism, and mitochondrial dysfunction associated with accumulated oxidative stress (Fig. 3). These metabolic alterations sustain not only cellular growth and survival but also actively promote virus-associated tumor development by fostering an immunosuppressive tumor microenvironment, driving chronic inflammation, and exacerbating genomic instability [144, 145].

#### Enhanced glycolysis and lactate accumulation mediate an immunosuppressive microenvironment

Multiple oncogenic viruses can induce host cells to undergo metabolic reprogramming similar to the Warburg effect, characterized by sustained enhancement of glycolysis under aerobic conditions and the production of large amounts of lactate. This process satisfies the energy and metabolic intermediate demands of viral replication and transformed cells, while also reshaping the local immune environment through lactate.

Oncoproteins and oncogenic viruses—including HPV E6/E7, EBV LMP1, KSHV vCyclin-mediated host carbamoyl-phosphate synthetase 2, aspartate transcarbamoylase, and dihydroorotase (CAD) hijacking, HBV HBx, MCPyV, and HIV—collectively modulate critical metabolic checkpoints such as glucose import (via GLUT1) and hexokinase (HK1) activity. These effectors drive glucose uptake by upregulating GLUT1, whereupon HK1 catalyzes the committed step of the Warburg effect (aerobic glycolysis), ultimately yielding lactate from pyruvate. Lactate efflux is then mediated by MCT1/MCT4 (with MCPyV regulating MCT1), thereby sculpting an immunosuppressive microenvironment [144–149]. Concurrently, the AARS1/AARS2 and cGAS-STING pathways regulate this process. Additionally, HIV Vpu modulates SNAT1 to influence alanine metabolism, while ALDH1L1 mediates folate metabolism to generate S-adenosylmethionine (SAM), further contributing to glucose metabolism reprogramming [150, 151]. HTLV-1 regulates Tap73 gene transcription via the HBZ protein, which binds to EZH2 to reduce its occupancy at the Tap73 promoter region. Concurrently, HBZ RNA activates Tap73 expression through the Batf3-IRF4 signaling pathway, thereby upregulating MCT1/4 expression levels and promoting lactate efflux. This process prevents the accumulation of cytotoxic substances within cells, indirectly promoting cancer cell growth [132].

Collectively, these findings demonstrate that viruses shape immunosuppressive tumor microenvironments by enhancing glycolysis and lactate metabolism, constituting a crucial component of their pro-cancer effects.

#### Rewiring lipid and cholesterol metabolism: membrane signaling platforms and viral replication advantages

Beyond glucose metabolism, the remodeling of lipid and cholesterol metabolism also constitutes a crucial component of virus-mediated metabolic reprogramming. Lipids serve not only as fundamental structural components of cell membranes and signaling platforms but also as essential materials for viral envelope formation and replication.

Lipid and cholesterol metabolism provides the structural foundation for viral replication, assembly, and release while simultaneously constructing membrane-associated signaling platforms that amplify proliferative and survival signals. During HPV infection, the E6 and E7 oncoproteins indirectly promote the maturation and activation of sterol regulatory element-binding proteins (SREBPs) by inhibiting p53 and pRB signaling pathways. This upregulates fatty acid synthase (FASN) and acetyl-CoA carboxylase (ACC) expression, enhancing fatty acid synthesis capacity [152]. EBV-encoded EBNA2 similarly synergizes with MYC to activate the SREBP pathway, promoting lipid synthesis and reshaping the tumor immune microenvironment through metabolic byproducts [144]. In hepatotropic viral infections, HBV and HCV activate metabolic master switches like HIF-1α and mTOR through their encoded proteins, upregulating lipid synthesis enzymes to induce hepatic lipid accumulation and steatotic changes, thereby creating a favorable metabolic environment for hepatocellular carcinoma development [145, 153]. In addition, the HIV-encoded Nef protein markedly enhances cholesterol synthesis and intracellular transport, thereby optimizing viral particle assembly and release efficiency while abnormally activating pro-cancer signaling pathways such as PI3K/Akt [120].

These studies demonstrate that the remodeling of lipid and cholesterol metabolism provides structural and signaling advantages for viral replication and persistent infection, exerting synergistic promotional effects during tumorigenesis.

#### ROS accumulation and mitochondrial dysfunction: DNA damage and chronic inflammation

Oxidative stress and mitochondrial homeostasis disruption constitute another key feature of virus-mediated metabolic reprogramming. Multiple viruses can induce excessive ROS production by regulating mitochondrial function, thereby exacerbating DNA damage, genomic instability, and chronic inflammatory responses.

In HPV-associated tumors, E5, E6, and E7 enhance mitochondrial ROS generation by modulating ROMO1 expression, promoting DNA damage and tumor progression [143]. Furthermore, HPV E6 disrupts mitochondrial metabolic homeostasis by reducing phosphorylated Akt levels, thereby regulating mitochondrial protein expression and cellular respiratory rates [154]. Other viral infections demonstrate similar logic. Although SARS-CoV-2 does not integrate into the host genome, non-structural protein 7 (NSP7) interacts with acyl-CoA dehydrogenase family member 3 (ACSL3) and thereby intersects with KRAS-driven metabolic pathways [155]. Meanwhile, ORF8 suppresses endoplasmic reticulum autophagy, induces ER stress, promotes formation of double-membrane vesicles, and degrades MHC-I molecules to achieve immune evasion [156, 157]. These processes bear striking similarities to the metabolic abnormalities and immune evasion mechanisms commonly observed in tumor cells.

The oncogenic importance of ROS accumulation and mitochondrial dysfunction lies in their ability to connect metabolism with genomic instability and inflammation. Excessive oxidative stress damages DNA, perturbs cellular signaling, and amplifies inflammatory responses, thereby making these processes particularly relevant to both tumor initiation and progression.

Collectively, multiple oncoviruses synergistically remodel host glycolysis, lipid metabolism, and mitochondrial function to establish a metabolic environment conducive to viral replication and tumorigenesis. These alterations not only provide energy and structural support for viral lifecycle progression and proliferating transformed cells but also profoundly shape immunosuppressive microenvironments through lactate accumulation, membrane signaling remodeling, and ROS–DNA damage axes, while promoting genomic instability. These mechanisms constitute core components of viral carcinogenesis and provide theoretical foundations for novel therapeutic strategies targeting metabolic–immune interactions.

### Role of viral infections in cancer: initiation, promotion, metastasis, and therapeutic resistance

Viral infections have been demonstrated as key drivers in the initiation and progression of multiple cancers, influencing the entire process from tumorigenesis and promotion to metastasis and treatment resistance. They continuously shape tumor biology in a stage-specific manner.

During early tumorigenesis, viruses largely persist as latent or chronic infections. Their primary role is not to directly drive malignant phenotypes, but rather to create a favorable environment for the gradual malignant transformation of normal cells by persistently inducing chronic inflammation, weakening apoptotic barriers, or disrupting genomic stability [158]. This low-intensity, long-term viral effect renders host cells more susceptible to crossing the threshold of carcinogenesis during repeated cycles of injury and repair [159]. HPV exemplifies this process: its E6 and E7 proteins inactivate the p53 and Rb signaling pathways, respectively, inducing genomic instability and dysregulating the cell cycle—thereby fueling the progression from cervical intraepithelial neoplasia to invasive cervical cancer [160]. Similarly, HBV exerts a well-defined initiating and driving role in hepatocarcinogenesis. HBx activates the NF-κB signaling pathway, induces chronic inflammatory responses, and under specific conditions directly triggers intrinsic malignant transformation of hepatocytes. HBx transgenic mice treated with DDC exhibit histological features of hepatocellular carcinoma and cholangiocarcinoma, further validating its critical role in tumorigenesis [161]. HCV, via its core protein, modulates HIF-1α, mTOR/AMPK pathways, and ROS production to remodel the hepatic metabolic environment, thereby creating conditions conducive to hepatocellular carcinoma development [145, 153, 162]. Furthermore, in MCPyV infection, viral genome integration and sustained expression of the oncogenic T antigen are considered decisive events in MCC development [138].

Upon entry into the promotion phase, the contribution of viruses to tumor progression undergoes a notable shift. By this point, tumor cells have already undergone full malignant transformation. Viruses no longer primarily exert their effects by inducing new genetic alterations but instead enhance tumor cell proliferation and survival advantages by maintaining continuous regulation of host signaling networks [162]. The persistent presence of viral-encoded products enables tumor cells to exhibit greater adaptability under endogenous inhibitory signals and exogenous therapeutic pressures [163]. In EBV-associated tumors, the virus significantly reduces cellular sensitivity to endogenous or therapeutically induced apoptotic signals by upregulating anti-apoptotic molecules such as Bcl-2 and Bcl-xL, thereby promoting tumor cell survival and proliferation [164]. KSHV similarly supports tumor promotion in immunocompromised hosts. In immunocompromised individuals, KSHV infection frequently shifts from latency to a pro-tumorigenic phase. Latent KSHV nuclear antigen (LANA) is detectable in over 90% of AIDS-associated KS patients, suggesting impaired immune surveillance is a critical prerequisite for KSHV-mediated tumor promotion [165]. Although HIV does not directly cause cancer, it indirectly promotes the proliferation of other virus-associated tumors by weakening host immune surveillance [166]. Furthermore, viruses such as HPV, HBV, HCV, and MCPyV similarly maintain cellular proliferative advantage or suppress apoptosis through various mechanisms [138, 153, 167, 168]. Thus, at this stage, viruses do not independently induce tumorigenesis but accelerate disease progression by sustaining tumor cell survival and amplifying pro-growth signals.

As tumors progress and acquire invasive and metastatic capabilities, viral infection increasingly remodels the tumor microenvironment and alters immune surveillance. Viruses indirectly facilitate tumor dissemination by altering the local immune landscape, promoting stromal remodeling, or enhancing the migratory capacity of tumor cells. While these effects do not directly cause metastasis, they facilitate the process by creating conditions permissive for tumor cell invasion and dissemination [169]. For instance, EBV-associated nasopharyngeal carcinoma spreads predominantly through the lymphatic system [164, 169], whereas KSHV promotes metastasis via HMGB1 upregulation and subsequent lymphatic dissemination [170]. Similarly, MCPyV underlies lymphatic metastasis in MCC [138], and HTLV-1 achieves systemic metastasis through hematogenous and lymphatic spread [171]. These phenomena underscore that during metastasis, interventions targeting virus-associated immune evasion or microenvironmental abnormalities may help reduce tumor progression and distant metastasis risks.

During the treatment-resistant phase, the impact of viral infection on tumor biology further manifests as regulation of therapeutic response. Studies indicate that virus-associated tumors exhibit reduced treatment response or drug resistance following radiotherapy, chemotherapy, or immunotherapy, potentially through enhanced tolerance to treatment-induced damage or interference with antitumor immune responses [169]. This resistance state typically exhibits dynamic and plastic characteristics, enabling tumors to progressively gain sustained survival advantages during repeated treatments. HPV E6/E7 proteins can weaken the DNA damage response, reducing sensitivity to radiotherapy and chemotherapy [172, 173]; EBV BHRF1 gene expression enhances cancer cell tolerance to chemoradiotherapy [174–176]; MCPyV-associated MCC patients exhibit primary or acquired resistance to immune checkpoint blockade therapy [139, 140]; HIV indirectly impacts tumor treatment efficacy through immunosuppression and antiviral resistance [141, 142]. In contrast, HBV, HCV, and KSHV also contribute to treatment resistance, but their effects depend on specific therapeutic regimens and are relatively limited. Therefore, identifying virus-associated resistance signatures and incorporating them into treatment decisions may offer novel entry points for overcoming therapeutic resistance.

Collectively, viruses systematically drive tumorigenesis, progression, metastasis, and treatment resistance by intervening in host cellular homeostasis at different stages through diverse mechanisms. Although the molecular pathways involved by different viruses exhibit significant differences, their common feature lies in persistently shaping a biological environment conducive to tumor evolution. An integrated understanding of these stage-specific roles not only deepens our comprehension of the biological essence of virus-associated tumors but also provides a theoretical foundation for developing stratified and combined therapeutic strategies based on viral characteristics.

## Tumor microenvironment and viral infection

The TME serves as the dynamic arena for tumorigenesis and progression, functioning as a complex ecosystem. It comprises diverse cellular components (cancer-associated fibroblasts, endothelial cells, immune cells such as T cells, B cells, and macrophages) alongside non-cellular constituents [177]. A growing body of research indicates that the tumor microenvironment plays a key active role in mediating tumor immune escape, promoting angiogenesis, accelerating invasion and metastasis, and inducing treatment resistance [178]. Through its pro-inflammatory, pro-fibrotic, immune evasion, immune exhaustion, and intercellular communication properties, tumors are able to survive and proliferate within the host.

From the earliest stages of tumorigenesis, the tumor microenvironment undergoes continuous evolution from the onset of tumorigenesis. When viral infection intervenes as a potent external stressor, it accelerates and reshapes this evolutionary process, transforming the normal tissue microenvironment into a malignant space more conducive to viral latency and virus-driven tumor progression [179]. Persistent viral infections can activate signaling pathways such as NF-κB and STAT3, promoting the release of inflammatory mediators such as IL-6, TNF-α, and IL-1β. This facilitates the recruitment of neutrophils, macrophages, and lymphocytes to the site of infection [180, 181]. This is manifested by enhanced cell proliferation [182], accumulation of DNA damage and mutations [183], and functional reprogramming of immune cells [169], thereby providing “oncogenic” signals that promote tumorigenesis. The natural outcome of chronic inflammation is often tissue fibrosis, one of the most prominent structural alterations in the tumor microenvironment. Virus-induced chronic inflammation and viral factors activate fibroblasts to differentiate into cancer-associated fibroblasts (CAFs), which secrete large amounts of extracellular matrix, forming a physical barrier to immune infiltration and drug delivery [184]. Together with immune evasion mechanisms, this reduces the efficiency with which cytotoxic T cells recognize and eliminate tumor cells, ultimately promoting the survival and proliferation of tumor clones [185]. Sustained viral antigen stimulation leads to T-cell exhaustion [186], upregulating inhibitory receptors such as programmed death receptor-1 (PD-1), T-cell immunoglobulin and mucin 3 (TIM-3), and lymphocyte activation gene 3 (LAG-3) [187, 188], resulting in a gradual loss of effector function until apoptosis occurs.

Viruses mediate intercellular communication via exosomes: infected cells release exosomes containing viral microRNAs, proteins, and genomic fragments [189]. Upon uptake by immune cells, CAFs, and endothelial cells, these exosomes induce functional reprogramming, amplifying the viral effect and enabling a small number of infected cells to exert global regulation over the entire microenvironment [190]. In summary, it is crucial to investigate the mechanisms by which viruses promote tumorigenesis through the remodeling of the tumor microenvironment. The mechanism can be summarized as follows: (Table 3).

**Table 3:** Mechanisms of viral oncogenesis through remodeling of the tumor microenvironment

Virus	Carcinogenic Mechanism Category	Core Mechanisms	Ref
HPV	Epithelium-Targeting Oncogenic Virus	Oncoprotein-driven transformation Immunosuppression Active remodeling of the TME	[6, 191–198]
MCPyV	Epithelium-Targeting Oncogenic Virus	Viral antigen-mediated cell cycle disruption Innate immune interference Modulating factor secretion	[199–204]
HBV	Inflammation-Fibrosis-Driven Virus	Inflammation-fibrosis-HCC progression Immune interference-induced exhaustion CAF activation and immunosuppression	[205–213]
HCV	Inflammation-Fibrosis-Driven Virus	HBV-like carcinogenic pathway Immune evasion and exhaustion Chronic inflammation-driven liver remodeling	[214–218]
EBV	Inflammation-Fibrosis-Driven Virus	Latent infection-driven oncogenesis Immune checkpoint upregulation Promotion of fibrosis and immunosuppression	[219–224]
KSHV	Inflammation-Fibrosis-Driven Virus	Secreted factor-mediated inflammation and angiogenesis Latency-associated immune evasion Construction of a pro-tumor network	[225–232]
HIV	Immune-Impairing & Amplifying Virus	Immune deficiency and chronic activation Direct depletion of CD4⁺ T cells Systemic disruption of the TME	[233–240]
HTLV-1	Immune-Impairing & Amplifying Virus	Chronic inflammation-driven leukemogenesis Viral protein-mediated dynamic immune evasion Vicious cycle-driven TME remodeling	[241–247]

TME, Tumor Microenvironment; HCC, Hepatocellular Carcinoma; CAFs, Cancer-Associated Fibroblasts

### Chronic inflammation and fibrosis as a carcinogenic niche

In virus-associated cancers, chronic inflammation does not simply reflect antiviral defense; rather, it creates a tissue context that favors DNA damage, abnormal proliferation, immune dysfunction, and progressive remodeling of the extracellular matrix [248]. In addition, fibrosis describes the pathological process in which excessive deposition of extracellular matrix components culminates in aberrant tissue structure and function. In the context of viral carcinogenesis, chronic inflammation triggered by infection and its terminal pathological alteration—tissue fibrosis—jointly constitute a critical “niche” driving the malignant transformation of normal cells. This niche provides essential conditions for tumorigenesis and progression by inducing genomic instability, promoting cell proliferation, suppressing immune surveillance, and altering the physicochemical properties of the matrix [249]. Viral carcinogenesis exhibits significant convergence, jointly shaping the pro-cancer microenvironment by driving persistent chronic inflammation and subsequent fibrotic remodeling. Based on their core pathogenic mechanisms, these processes can be categorized into three representative types.

#### Epithelial-associated carcinogenic viruses and their signal reprogramming characteristics

Epithelial-associated viruses directly target epithelial cells, driving carcinogenesis by establishing latent infections and sustained immune activation [124]. In this setting, infected epithelial cells are not merely passive viral reservoirs; rather, they become active sources of inflammatory mediators, angiogenic factors, and stromal signals that progressively transform the surrounding tissue into a niche permissive for carcinogenesis.

HPV is the clearest example of this pattern. HPV infection represents one of the most prevalent sexually transmitted infections worldwide and imposes a substantial cancer burden [6]. HPV disrupts the normal immune function of basal epithelial cells by expressing oncoproteins such as E6 and E7, thereby inducing persistent infections that create conditions conducive to carcinogenesis [191]. To clear the virus, the immune system initiates a persistent response; however, when the virus successfully evades clearance, this process transforms into a chronic low-grade inflammatory state. With time, accumulated genetic and epigenetic abnormalities precipitate intraepithelial neoplasia, which may progress invasive carcinoma [192]. Chronic inflammation serves as a key driver in the progression of cervical precancerous lesions. Through multi-omics analysis, Ilhan et al. constructed a microbe-host co-metabolism network, revealing that HPV infection induces local microbiome imbalance and metabolic remodeling. The reciprocity between these two processes exacerbates chronic inflammation, culminating in uncontrolled cell proliferation and malignant transformation. These findings provide additional evidence for the central mechanism by which HPV infection propagates chronic inflammation [193]. This mechanistic research reveals the “upstream” pathway from infection to carcinogenesis, suggesting that interventions must extend beyond simple antiviral approaches to simultaneously target the inflammatory microenvironment and modulate the microbiota and metabolism. Furthermore, clinical studies indicate that the persistent presence of such chronic inflammation can trigger tissue remodeling responses such as fibrosis, which is significantly associated with more aggressive pathological features and poor prognosis in patients [194].

Beyond HPV, the MCPyV virus is another typical representative in this category. This virus typically lies dormant within host skin or hair follicle basal cells, subsequently integrating its genome into host DNA. It disrupts host cell cycle regulation and immune surveillance by expressing viral early antigens [199]. Viral antigen expression induces cellular DNA damage responses, oxidative stress, and chromosomal instability, accompanied by stromal cell activation and the emergence of fibrotic phenotypes [200]. Thus, epithelial-associated viruses demonstrate how localized epithelial reprogramming can extend outward into a broader protumor ecosystem.

#### Inflammation-fibrosis-driven carcinogenic viruses and chronic microenvironmental remodeling

Viruses that drive inflammation and fibrosis primarily target parenchymal organs; their oncogenic potential is tightly coupled with the progressive tissue fibrosis that results from chronic inflammation [206]. Furthermore, in this model, the driving force behind malignant transformation stems primarily from long-term ecological remodeling resulting from repeated cycles of tissue damage and repair.

Taking HBV as an example, the persistent cycle of liver inflammation and repair damage it induces represents the core pathway driving the progression of liver tissue from fibrosis and cirrhosis to hepatocellular carcinoma [205, 206]. Clinical studies have confirmed that the degree of liver fibrosis serves as a critical independent predictor of postoperative recurrence in patients with HBV-associated hepatocellular carcinoma [207]. Furthermore, HCV follows a closely parallel pathway of chronic inflammation, fibrosis, and carcinogenesis [211, 213].

Notably, although EBV infects epithelial cells, its oncogenic effects are mediated largely through chronic inflammatory signaling, immune modulation, and remodeling of the microenvironment. EBV establishes a latent infection, evades immune surveillance, and provokes persistent chronic inflammation. This inflammatory milieu triggers genomic instability via the release of cytokines, ultimately fostering tumorigenesis [219]. Furthermore, EBV infection induces fibroblast-to-myofibroblast transformation by activating signaling pathways such as transforming growth factor-β, thereby upregulating collagen deposition. Concurrently, the virus releases exosomes carrying EBV-encoded LMP1, activating the Yes-associated protein 1 (YAP1)/fibroblast activation protein alpha (FAPα) signaling axis in adjacent fibroblasts, thereby fostering a fibrotic microenvironment [220]. Beyond these viruses, KSHV similarly promotes tumor microenvironment remodeling through inflammatory cytokine secretion, angiogenesis, and immune regulation, exhibiting highly overlapping mechanisms with inflammation-fibrosis-driven viruses [225, 226].

In summary, future prevention and treatment strategies should not be limited to mere antiviral control but should shift toward multifaceted interventions that combine pathogen clearance, resolution of inflammation, control of fibrosis, and restoration of tissue homeostasis.

#### Immunodeficiency-enhancing viruses and tumor-promoting effects

Immunodeficiency-promoting viruses primarily create conditions conducive to tumorigenesis by disrupting the host's immune homeostasis [233, 234]. In these cases, the carcinogenic microenvironment is determined not only by local fibrosis or epithelial remodeling, but also by defects in systemic or tissue-specific immune balance.

HIV is a classic example of this category. The systemic acquired immunodeficiency and chronic systemic immune activation it induces significantly increase patients' risk of developing multiple malignancies. Studies indicate that even under effective antiviral therapy, elevated levels of inflammatory markers (such as IL-6) in HIV-infected individuals remain independently associated with increased cancer risk [235]. Concurrently, immune dysregulation—particularly in the context of co-infections—can drive tissue remodeling processes like liver fibrosis, accelerating disease progression [250]. Thus, HIV-driven tumor promotion depends on a dual process of immune deficiency and chronic inflammatory amplification.

HTLV-1 represents a distinct destructive pattern: it does not cause widespread immunodeficiency but instead induces intense chronic inflammation, creating severe local immune dysregulation (e.g., in the central nervous system or blood microenvironment) and activating pro-fibrotic pathways like TGF-β, collectively driving the development of adult T-cell leukemia [241, 242]. Accordingly, immune-perturbing viruses broaden the concept of the carcinogenic niche beyond tissue fibrosis alone, demonstrating that chronic inflammatory damage may also be propagated through systemic or hematologic immune disequilibrium.

**Fig. 4:**
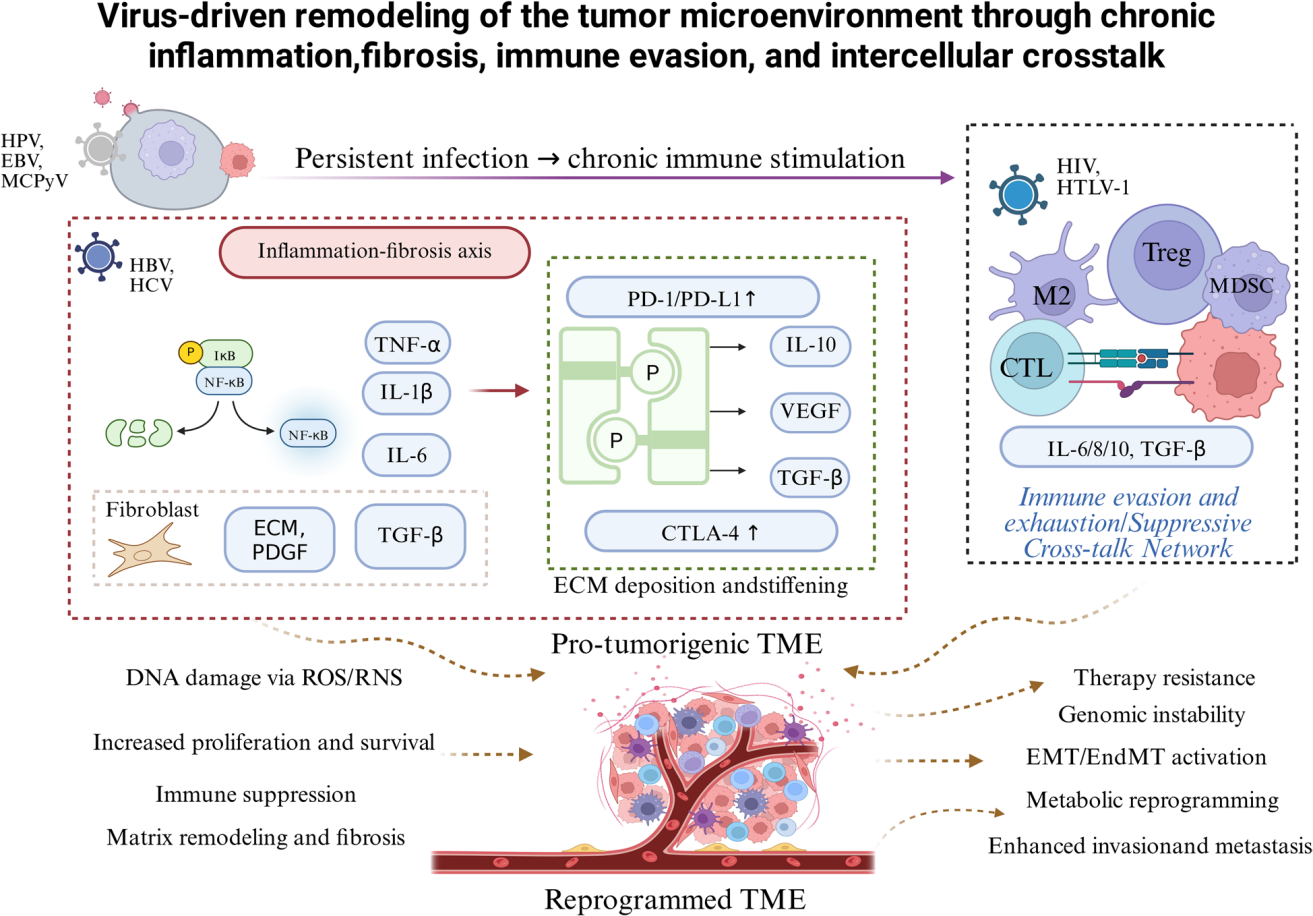
Integrated mechanisms by which chronic viral infection remodels the tumor microenvironment. Persistent or latent viral infection imposes sustained immune stimulation that chronically activates inflammatory signaling pathways, particularly the NF-κB and STAT3 axes, in infected epithelial or parenchymal cells. NF-κB activation promotes continuous production of pro-inflammatory cytokines, including TNF-α, IL-1β, and IL-6, whereas STAT3 signaling induces immunosuppressive and pro-fibrotic mediators such as IL-10, TGF-β, and VEGF. The convergence of these pathways establishes a chronic inflammatory state characterized by excessive reactive oxygen and nitrogen species (ROS/RNS), leading to cumulative DNA damage, genomic instability, and enhanced survival and proliferation of transformed cells. Sustained inflammation further activates resident fibroblasts and drives their differentiation into cancer-associated fibroblasts, resulting in excessive extracellular matrix deposition, matrix remodeling, and tissue fibrosis, which together form a physical and biochemical barrier that limits immune cell infiltration and therapeutic delivery. In parallel, cytokine-mediated intercellular crosstalk reshapes the immune compartment, promoting macrophage polarization toward an M2-like phenotype, expansion of regulatory T cells, functional impairment of cytotoxic T lymphocytes, and upregulation of immune checkpoint pathways such as PD-1/PD-L1 and CTLA-4. Through continuous signaling exchange among infected tumor cells, stromal cells, endothelial cells, and immune cells, these inflammatory, fibrotic, and immunosuppressive processes reinforce one another, ultimately reprogramming the tumor microenvironment to favor immune evasion, EMT/EndMT activation, metabolic reprogramming, enhanced invasion and metastasis, and resistance to therapy

These mechanistic studies offer clear directions for interventions aimed at preventing and treating virus-associated tumors. First, disrupting the “inflammation-fibrosis” axis is a central priority. Developing agents that block key mediators—such as TGF-β and IL-6—or signaling pathways including NF-κB and STAT3 could delay or reverse the establishment of a carcinogenic microenvironment [220, 251]. Second, in the context of certain viruses, modulating the local ecological imbalances they induce (e.g., HPV-associated microbiota and metabolic remodeling) may represent novel intervention strategies [193]. Furthermore, fibrosis represents both a critical clinical biomarker and a viable therapeutic target. For instance, incorporating liver fibrosis severity (FIB-4 index) into risk stratification and monitoring systems for HBV/HCV-infected individuals enables early warning and personalized management [207, 252]. Similarly, reversing fibrosis associated with cervical cancer may improve patient prognosis [194]. Finally, for viruses like HIV that induce systemic immune dysregulation, early and effective antiviral therapy to restore immune homeostasis is fundamental to reducing overall tumor risk [235]. In summary, future prevention and treatment strategies should transcend mere antiviral approaches, shifting toward multidimensional integrated interventions that combine “pathogen clearance, inflammation resolution, fibrosis reversal, and homeostasis restoration.” This holistic approach fundamentally dismantles the ecological niche upon which viruses rely to promote carcinogenesis (Fig. 4).

### Viral strategies of immune evasion and immune exhaustion

The host immune system recognizes and eliminates invading viruses via both innate and adaptive immune pathways. However, chronic or latent viral infections induce multi-level immune escape mechanisms, leading to the gradual failure of the host immune response and subsequently triggering persistent infection and immune exhaustion [253]. Immune evasion encompasses the strategies by which viruses escape host immune recognition, including interference with antigen presentation, suppression of interferon signaling, and remodeling of the immune microenvironment [254]. Immune evasion enables viruses and infected cells to avoid immune recognition through interference with antigen presentation, suppression of interferon signaling, disruption of innate immune sensing, and remodeling of the local immune microenvironment [255]. Once persistence is established, chronic antigen exposure progressively drives immune exhaustion, a dysfunctional state characterized by reduced effector activity and sustained expression of inhibitory receptors such as PD-1, cytotoxic T lymphocyte-associated antigen 4 (CTLA-4), TIM-3, and LAG-3 [256].

Importantly, immune exhaustion does not occur in isolation. Exhausted T cells synergize with regulatory T cells (Tregs), myeloid-derived suppressor cells (MDSCs), and other inhibitory cells/receptors to form complex immunosuppressive networks, further exacerbating T cell dysfunction [257]. Thus, the virus-associated TME should be understood not as a collection of separate immune defects, but as an integrated immunosuppressive system that sustains viral persistence, weakens tumor clearance, and promotes disease progression [258]. Therefore, a detailed understanding of virus-mediated immune evasion and exhaustion is essential for the rational design of targeted immunotherapeutic strategies.

Different viral categories synergistically drive this cycle through specific molecular strategies. Among epithelial-associated viruses, HPV evades immune recognition through low-level expression of oncoproteins and actively secretes factors like IL-10 and TGF-β to establish local immunosuppression. These cytokines directly suppress the antitumor activity of CD8⁺ T cells and natural killer cells while further inducing the recruitment and activation of immunosuppressive cells, creating a vicious cycle [195]. Consistent with this logic, a systematic review indicates that HIV-infected individuals or patients receiving immunosuppressive therapy exhibit significantly elevated risks of persistent HPV infection and progression to cervical intraepithelial neoplasia (CIN), with hazard ratios of 2.20 and 1.33, respectively [196]. MCPyV employs a related strategy: its large T antigen (LT-ag) helps maintain latency by suppressing expression of viral DNA replication-associated genes, evading immune recognition; while the small T antigen (sT-ag) interferes with innate immune signaling pathways, such as suppressing NF-κB transcriptional activity and weakening antiviral cytokine production, ultimately creating decisive conditions for virus-driven tumorigenesis and progression [201].

HBV, a virus classically driven by inflammation and fibrosis, utilizes a comparatively direct mechanism of immune escape. For instance, viral proteins including HBx degrade or interfere with key innate immune signaling molecules such as RIG-I and STAT1 [208], effectively blunting the interferon response. Prolonged exposure to viral antigens within a sustained inflammatory milieu progressively depletes virus-specific CD8⁺ T cells [209]. HCV shares similar immune evasion mechanisms with HBV [214, 215]. Epidemiological data reveal that HIV-infected individuals face a 2.79-fold increased risk of hepatocellular carcinoma (HCC) compared to the general population, highlighting the amplifying effect of immune deficiency on HBV-mediated carcinogenesis [210].

EBV and KSHV also illustrate a similar strategy. EBV exploits latent proteins (e.g., LMP1) to suppress antigen presentation and upregulate PD-L1, thereby evading T cell surveillance [221]. Consistent with the virus's capacity for sustained immunosuppression, clinical evidence points to higher PD-L1 expression and more pronounced CD8⁺ T cell exhaustion in nasopharyngeal carcinoma tissues obtained from regions with elevated EBV prevalence [222]. KSHV likewise suppresses antigen-presentation pathways, disrupts interferon signaling and NF-κB activation, and promotes the release of immunosuppressive cytokines, ultimately accelerating the formation of an immune escape microenvironment [227].

Viruses that disrupt immune homeostasis systematically amplify this process. HIV directly causes acquired immunodeficiency by destroying CD4⁺ T cells, representing the most extreme form of systemic immune evasion. The resulting chronic systemic immune activation not only induces abnormal T cell activation and exhaustion but also provides a foundation for the evasion and tumorigenesis of other oncogenic viruses (such as KSHV) [210]. A prospective cohort study confirmed that among HIV-infected individuals receiving sustained antiviral therapy, each doubling of the inflammatory marker IL-6 levels corresponded to an approximately 38% increase in cancer risk [235]. Conversely, HTLV-1 initially elicits immune recognition via the potent immunogenicity of its Tax protein but subsequently leverages the HBZ protein to dampen host responses and establish latency [243]. This dynamic interplay ultimately exhausts virus-specific T cells and fosters an immunosuppressive microenvironment replete with PD-L1 [244]. Clinical observations reveal that in immunocompromised populations, both the incidence and progression rate of HTLV-1-associated leukemia (ATLL) are significantly increased [245]. Similar mechanisms are widely observed in KSHV [169, 227], HCV [211, 212], and MCPyV [201, 202].

The shared “escape-exhaustion” common axis described above offers distinct and actionable targets for immunotherapy in virus-associated malignancies. Therapeutic targeting of immune checkpoints represents a direct approach for reversing T-cell exhaustion. Given that the PD-1/PD-L1 pathway serves as a shared exhaustion hub for multiple viruses including EBV, HPV, and HTLV-1 [202, 221, 244], checkpoint inhibitors hold broad application potential. In parallel, therapies targeting virus-specific immune-disrupting proteins, such as HBx or LMP1, may weaken the source of immune suppression itself. Combination strategies that reshape the virus-induced immunosuppressive microenvironment are equally important. These include TGF-β inhibition, depletion of regulatory T cells, or other interventions that restore antitumor immunity [195]. Moreover, for viruses causing systemic immune deficiency like HIV, early highly effective antiviral therapy to restore immune homeostasis is fundamental to preventing all subsequent virus-associated malignancies. Overall, the most effective strategy is likely to be an integrated immune intervention that combines blocking escape, reversing exhaustion, and reshaping the microenvironment to interrupt the vicious cycle established by persistent viral infection.

### Crosstalk between infected cells within tumor microenvironment

In virus-associated tumors, infected cells do not exist in isolation but actively orchestrate sustained intercellular crosstalk within the TME. Through persistent release of viral nucleic acids, cytokines, metabolites, and extracellular vesicles, infected cells engage immune, stromal, and vascular compartments in a coordinated manner. Viral pathogen-associated molecular patterns (PAMPs) continuously activate innate immune sensors such as Toll-like receptors (TLRs) and RIG-I/MDA5, establishing a low-grade yet persistent inflammatory state [259, 260]. Recurrent immune cell infiltration, together with repeated exposure to inflammatory cytokines and proteases, drives cycles of tissue injury and repair, amplifying inflammatory signaling and progressively reshaping the local tissue architecture [261].

Within this chronically inflamed milieu, profibrotic and angiogenic factors including TGF-β, IL-6, VEGF, and PDGF activate endothelial cells, pericytes, mesenchymal stem cells, and fibroblasts, inducing their phenotypic reprogramming. This process promotes extracellular matrix remodeling, vascular dysfunction, and the establishment of an immunosuppressive microenvironment characterized by aberrant angiogenesis and impaired immune surveillance [262, 263]. Concurrently, persistent viral antigen stimulation and immune regulatory remodeling drive immune cell populations—including CD8⁺ and CD4⁺ T cells, regulatory T cells, natural killer cells, dendritic cells, and myeloid-derived suppressor cells—toward exhausted, suppressed, or protumor phenotypes [169, 186, 264]. Collectively, these processes form an interconnected inflammatory–fibrotic–immunosuppressive network that constitutes a common biological framework sustaining viral persistence, immune evasion, and tumor progression [169, 265, 266].

Although oncogenic viruses target distinct cell types, their remodeling of the tumor microenvironment converges on a limited number of dominant signaling logics. Epithelial-associated viruses, such as high-risk HPV and MCPyV, primarily reshape the microenvironment through virus-driven epithelial reprogramming. Viral oncoproteins, exemplified by HPV E6/E7, induce sustained inflammatory signaling and selectively activate endothelial cells, macrophages, and cancer-associated fibroblasts via angiogenic and chemokine pathways. This results in extracellular matrix deposition, vascular abnormalities, and progressive CD8⁺ T cell dysfunction, forming a mechanically rigid and immunosuppressive niche that facilitates tumor invasion and immune escape [197, 198, 203, 204].

Viruses characterized by inflammation–fibrosis coupling, notably HBV and HCV, exemplify chronic immune-mediated microenvironmental remodeling. Persistent PAMP-driven innate immune activation maintains long-term hepatic inflammation, while infected hepatocytes continuously secrete profibrotic mediators such as TGF-β and PDGF during repeated cycles of injury and repair. These signals drive fibroblast-to–cancer-associated fibroblast transition, extracellular matrix remodeling, and immune checkpoint enrichment, jointly promoting immune suppression and hepatocellular carcinoma development [211–213].

EBV- and KSHV-associated tumors exhibit marked immune evasion accompanied by stromal reprogramming. EBV latent proteins, particularly LMP1, activate the NF-κB and JAK-STAT signaling pathways to induce PD-L1 expression and recruit immunosuppressive immune subsets, thereby attenuating cytotoxic immune responses [222, 223]. At the same time, virus-infected tumor cells reprogram fibroblasts to adopt cancer-associated phenotypes, thereby increasing collagen deposition, matrix stiffeness, and tissue tension. These changes restrict immune cell infiltration while facilitating tumor cell migration and dissemination, generating a microenvironment permissive to viral persistence and tumor progression [220, 224, 228–232].

Viruses that disrupt immune homeostasis, including HIV and HTLV-1, indirectly remodel the tumor microenvironment by inducing chronic interferon signaling and widespread immune exhaustion. Sustained type I interferon activation, combined with enhanced inhibitory ligand–receptor interactions, drives profound dysfunction of T cells and natural killer cells while promoting fibroblast activation and extracellular matrix accumulation. This immunosuppressive and fibrotic microenvironment markedly increases susceptibility to virus-associated malignancies and facilitates disease progression [236, 240, 246, 247].

In summary, viral infection comprehensively remodels the tumor microenvironment through multiple mechanisms including chronic inflammation, immunosuppression, and intercellular signaling (Fig. 4). These interconnected processes not only sustain viral persistence but also promote cellular malignant transformation and induce therapeutic resistance. Deepening our understanding of the interactions between viruses and the tumor microenvironment will provide novel strategies for immunotherapy and metabolic interventions in virus-associated tumors.

## Therapeutic and preventive strategies

With the rapid advancement of modern medicine, intervention strategies for virus-associated cancers are gradually shifting from traditional broad-spectrum treatments to precision targeted therapies. Given that viral infection is the primary pathogenic factor in the development of virus-associated malignancies, vaccination and antiviral treatments have become key preventive measures. Concurrently, immunotherapies and cell therapies targeting viral antigens or host carcinogenic pathways offer new avenues for patient care [267, 268].

### Antiviral therapies and their role in cancer prevention

Prevention is the most economical, effective, and sustainable means of controlling virus-induced cancers. Vaccination confers protection against viral infections, and antiviral therapy clears existing chronic infections. Together, these interventions markedly reduce the risk of specific cancers and correlate with better long-term survival among patients with virus-related malignancies. Moreover, directly targeting virus-specific oncoproteins or host carcinogenic pathways hijacked by viruses offers novel approaches for chemoprevention and early intervention in cancer.

#### Vaccine therapy

Vaccination is the most cost-effective preventive measure against virus-associated tumors by inducing specific immune responses that block viral adhesion and invasion of host cells. The large-scale application of HPV and HBV vaccines has become a model. Currently available HPV and HBV preventive vaccines have significantly reduced the incidence of cervical cancer and hepatocellular carcinoma, establishing a benchmark for vaccine-based treatment of cancer caused by viral infections [269–273].

Current prophylactic HPV vaccines consist of virus-like particles assembled from recombinant L1 proteins. By inducing high-titer neutralizing antibodies that block viral entry, they effectively prevent HPV infection establishment [274]. Next-generation multivalent vaccines now offer protection against more than 90% of high-risk HPV subtypes [275]. However, existing HPV vaccines provide only prophylactic benefits and do not treat established infections [275]. A recent analysis suggests that, relative to placebo, investigational therapeutic HPV vaccines lower the overall incidence of cervical intraepithelial neoplasia (precancerous lesions caused by HPV infection) by 22.1%, but the overall response rate for complete lesion clearance is only 23.6% [276]. Although no therapeutic vaccines for cervical cancer have yet to receive market approval, research in this area continues to advance steadily. The widely used HBV vaccine serves a prophylactic role. A randomized controlled trial conducted in China confirmed that by inducing specific humoral immunity, it can achieve a 70% protective rate against the risk of hepatocellular carcinoma mortality [271].

Despite the significant efficacy of preventive vaccines, vaccines targeting EBV, KSHV, HCV, and others remain in the research and development phase. Core bottlenecks include the complex mechanisms of viral infection and the lack of clarity regarding key antigen targets [277]. Current development strategies have progressively shifted toward multivalent antigen design. This approach simultaneously targets multiple key antigens associated with viral invasion or latent infection, thereby concurrently activating humoral and cellular immunity to enhance the broad-spectrum protective efficacy of vaccines [19, 20, 37, 278–280]. For example, KS virus can establish lifelong latent infection with restricted viral gene expression during latency. Therefore, employing a multivalent antigen strategy to simultaneously present lytic-phase glycoproteins (gpK8.1, gB, gH/gL) and latent-phase antigens (LANA) to induce broad humoral and cellular immune responses is of critical importance [37, 38]. Vaccine development targeting other oncogenic viruses is also actively advancing (Table 1).

#### Targeting viral and host carcinogenic pathways

Viral carcinogenesis depends on the interaction between viral proteins and host signaling pathways. The most effective strategy involves simultaneously targeting critical stages of the viral life cycle and the hijacked host pathways. Both approaches—directly eliminating viral genomes via gene editing technologies and intervening in virus-hijacked host pathways (such as DNA repair, immune responses, cell proliferation, and metabolic pathways) using small-molecule inhibitors or immunomodulators—demonstrate significant potential for clinical application (Table 2).

### Biomarker discovery, diagnostic, prognostic and predictive implications

Biomarkers serve as the cornerstone of virus-associated cancer research and play a pivotal role throughout disease management. Existing studies confirm that biomarkers primarily encompass four major systems: viral nucleic acids and their oncoproteins, host genes and epigenetic alterations, tumor microenvironment characteristics, and multi-omics integrated markers [85]. These markers not only facilitate early tumor screening, diagnostic staging, and progression risk prediction but also provide critical guidance for treatment efficacy assessment and long-term prognosis evaluation.

#### Discovery of biomarkers

Virus-associated tumors provide ideal models for biomarker exploration, primarily due to their unique etiological mechanisms. These malignancies are typically driven by a single oncogenic virus (e.g., EBV or HPV), with their carcinogenesis relying on relatively stable and singular viral oncogenic pathways. This provides a clear molecular basis for screening stable, specific biomarkers [281]. The persistent presence and expression of viruses within tumor cells render their genomic DNA, messenger RNA, or viral proteins (such as E6/E7 proteins) highly specific and readily detectable molecular markers [92]. In parallel, viral infection remodels the tumor microenvironment, triggering specific metabolic reprogramming, immune responses, and chronic inflammatory reactions. This leads to detectable characteristic alterations in host metabolism, immunity, and inflammation [169]. These markers play a crucial role in diagnosis, prognosis, and predicting treatment response.

#### Diagnostic significance of biomarkers

The core value of biomarkers lies primarily in enabling accurate diagnosis throughout the progression from viral infection to precancerous lesions and ultimately tumor formation. Essentially, they serve as a critical bridge, translating the abstract, virus-driven carcinogenic process into concrete, clinically identifiable molecular or histological signals.

Viral nucleic acids and oncoproteins provide the most specific markers for etiological confirmation. For instance, HPV E6/E7 mRNA detection precisely reflects high-risk infection status, plasma EBV DNA testing has become a core tool for early nasopharyngeal carcinoma screening, while HBV DNA and HCV RNA detection distinguish between chronic infection and different disease stages such as cirrhosis and early hepatocellular carcinoma [282–284]. Other viruses, including KSHV, MCPyV, and HTLV-1 have their own distinct histological markers [285–288]. The detection of viral nucleic acids and viral proteins not only clarifies causal associations but also reflects the intensity of viral carcinogenic activity. This extends to indirect surrogate markers reflecting persistent viral interference with host cells, such as impaired tumor suppressor gene function, abnormal epigenetic regulation, and the restructuring of key metabolic and signaling networks. Ultimately, it expands to host response markers at the tissue and systemic levels, including fibrosis severity, immune infiltration patterns, and alterations in tumor microenvironment structure.

Molecular alterations in host cells induced by persistent viral infection provide critical evidence for grading lesions. For instance, p16 protein overexpression serves as a surrogate marker for HPV E7-mediated inactivation of the retinoblastoma protein pathway; Methylation of the MAL and EPB41L3 genes in HPV-associated lesions [289], and methylation of the RASSF1A and GSTP1 genes in HBV/HCV -associated hepatocellular carcinoma, can assess progression risks in virus-related cancers. Meanwhile, liver fibrosis scoring systems effectively evaluate the “inflammation-fibrosis-carcinogenesis” progression [289–292]. Furthermore, tumor microenvironment markers such as PD-L1 expression and regulatory T cell (Treg) enrichment can further assist in tumor subtyping, providing reference for subsequent therapeutic strategy selection [169].

#### Prognostic significance of biomarkers

The prognostic assessment of tumor-associated viruses requires multidimensional analysis of core indicators, including viral load dynamics, oncoprotein expression levels, the immune microenvironment, and fibrosis characteristics. Fluctuations in viral load reflect tumor biological behavior and serve as a basis for prognostic stratification. For instance, in patients with nasopharyngeal carcinoma, both baseline EBV DNA levels and their longitudinal dynamics are predictive of recurrence and metastasis risk [293]. Similarly, a high HBV viral load significantly increases the incidence and mortality of hepatocellular carcinoma [294], while persistent HCV RNA positivity portends a greater likelihood of liver fibrosis progression and hepatocarcinogenesis [295]. In contrast, individuals with HPV-positive head and neck cancer patients exhibit superior clinical outcomes than their HPV-negative counterparts—an advantage largely ascribed to enhanced immune activity within the tumor microenvironment [296].

Second, the expression levels of viral oncoproteins carry clear prognostic significance. For example, EBV LMP1, HPV E6/E7 proteins, MCPyV large T antigen, and HTLV-1 Tax/HBZ proteins are all closely associated with tumor invasiveness, proliferative capacity, and metastatic risk [85, 297, 298].

Furthermore, tumor immune microenvironment characteristics and tissue fibrosis severity jointly determine prognostic outcomes. High infiltration of CD8⁺ tumor-infiltrating lymphocytes in tumor tissues typically indicates survival benefit [299], whereas regulatory T cell enrichment and high expression of T cell exhaustion markers suggest immune escape and poor prognosis [300]. The staging of HBV/HCV -associated liver fibrosis is also closely linked to hepatocellular carcinoma incidence and patient survival rates [301]. Comprehensive evaluation of these indicators provides critical evidence for prognosis assessment and personalized treatment strategy development in virus-associated tumors.

Beyond traditional prognostic markers, metabolic reprogramming and phase separation alterations have emerged as highly promising novel prognostic indicators in oncology. Different viruses drive tumorigenesis by specifically inducing metabolic abnormalities in tumor cells: HPV and EBV cause lactate accumulation and enhance glycolytic pathway activity; HCV and HBV primarily trigger intracellular lipid droplet accumulation and lipid metabolism disorders; HTLV-1 and EBV also induce excessive ROS accumulation and mitochondrial dysfunction.

These virus-mediated metabolic abnormalities are not only directly linked to tumor cell invasion and metastasis capabilities but also induce immune cell exhaustion by reshaping the tumor microenvironment. The associated metabolic phenotypes have been validated as effective emerging indicators for assessing tumor malignancy and patient prognosis.

#### Predictive significance of biomarkers

Predictive biomarkers provide critical support for developing personalized treatment plans, playing a central role particularly in selecting combination therapy strategies. With the application of machine learning technologies, predictive models integrating multi-omics data, imaging features, and clinical information have significantly improved the accuracy of predicting treatment response and recurrence risk [302].

Established predictive models have identified viral nucleic acids (e.g., HPV DNA, EBV DNA), host oncogenes (e.g., PIK3CA, TP53), immune checkpoint molecules (e.g., PD-L1), and epigenetic features (e.g., DNA methylation) may represent core predictive targets [303–306]. Models constructed using algorithms such as extreme gradient boosting, random forests, and recurrent neural networks not only enable early prediction of lesion progression risk but also enhance predictive efficacy through strategies like ensemble learning [307–309]. However, substantial modeling hurdles remain for certain virus-associated malignancies, particularly those linked to KSHV. Limited follow-up cohort data, high clinical subtype heterogeneity, and lack of standardized biomarker detection platforms hinder the development of high-quality multimodal models. Advancing research in this domain will depend on the adaptation of modeling insights and methodological frameworks derived from other virus-associated tumor types [310–312].

Similarly, machine learning algorithms have been applied to biomarker discovery and prognostic prediction in HIV-associated tumors. In a cohort of HIV-Mycobacterium tuberculosis co-infected patients, Sun et al. developed an interpretable machine learning model using lightweight gradient-boosted decision trees, SMOTE oversampling, and SHAP analysis. Inputting markers like CD4 and CD8, the model achieved an area under the curve exceeding 0.77 for predicting treatment failure or mortality risk, demonstrating robust predictive capability in complex comorbid scenarios [313].

The biomarker systems for virus-associated tumors are evolving from traditional approaches combining viral nucleic acid detection with single host markers toward multidimensional integrated frameworks incorporating genomic, epigenetic, metabolic reprogramming, immune microenvironment, and phase separation characteristics. These models not only significantly enhance predictive accuracy for treatment response, recurrence risk, and patient survival—enabling more precise risk stratification—but also reveal key biomarkers and pathways driving disease progression through interpretability analysis. This provides novel insights into elucidating viral carcinogenesis mechanisms and developing precision treatment strategies.

### Precision oncology therapies

Virus-associated tumors exhibit virus-mediated immune response-driven characteristics during malignant transformation, making them highly susceptible to developing resistance to single-agent therapies. In precision oncology, combining traditional surgery, radiotherapy, and chemotherapy with immune checkpoint blockade therapy, chimeric antigen receptor T-cell immunotherapy, RNA-targeted therapy, and oncolytic virus therapy can overcome resistance through synergistic effects and enhance treatment efficacy.

#### ICB combination therapies

Immune checkpoint blockade therapy typically reactivates antitumor immune function by blocking inhibitory signaling pathways such as CTLA-4 and PD-1/PD-L1, thereby liberating T cells from immunosuppression [314]. However, monotherapy is constrained by intrinsic tumor resistance mechanisms and immunosuppressive microenvironments, leading to significant interindividual variability in efficacy. Consequently, combination regimens with conventional chemoradiotherapy, anti-angiogenic therapy, or dual immune checkpoint blockade have become the mainstream approach. Radiotherapy and chemotherapy can enhance the therapeutic response to immune checkpoint inhibitors through mechanisms such as direct killing of tumor cells releasing antigens, reducing infiltration of immunosuppressive cells, and inducing immunogenic cell death. Improved overall and progression-free survival has been documented in multiple virus-associated tumors treated with such combinations [315–318]. For instance, the addition of pembrolizumab to concurrent chemoradiotherapy raised the 2-year progression-free survival rate to 68% in high-risk locally advanced cervical cancer patients [319]. Similar conclusions have been drawn from other viral studies [11, 320–325]. Among these, avelumab has become the first drug approved for metastatic MCC. Studies show that avelumab combined with chemotherapy significantly increases the proportion of activated dendritic cells in patients compared to pre-chemotherapy levels [326]. The role of immune checkpoint blockade in HIV infection remains controversial [327, 328].

Dual immune checkpoint blockade strategies aim to overcome immune tolerance by synergistically disrupting distinct signaling pathways. For instance, blocking PD-1 in conjunction with CTLA-4, LAG-3, and TIGIT has yielded superior clinical outcomes in melanoma as well as select virus-associated tumors [329–334]. Additionally, the combination of immune checkpoint inhibitors with therapeutic vaccines or T-cell agonists enhances treatment efficacy through synergistic effects of actively inducing specific immune responses and lifting immunosuppression [335–337]. It is important to note that different virus-associated tumors exhibit varying responses to immune checkpoint blockade therapy. Some tumors remain resistant to effective immunotherapy due to constraints imposed by their unique immune microenvironment characteristics. Furthermore, combination therapies may induce immune-related adverse events. Addressing these challenges requires in-depth exploration of viral carcinogenesis mechanisms and the regulatory patterns of the immune microenvironment. For example, HTLV-1-associated adult T-cell leukemia/lymphoma currently lacks effective immunotherapy options, and PD-1 inhibitors may accelerate disease progression, limiting their clinical application [338].

Significant variations exist in the response levels to PD-1/PD-L1 inhibitors among different virus-driven malignancies. Moreover, immune-related adverse events induced by combination therapies cannot be overlooked. Future research should continue to delve into viral carcinogenesis mechanisms and, based on this foundation, develop personalized immunotherapy regimens targeting specific viruses to advance the field of immunotherapy for virus-associated cancers.

#### Combination applications of CAR-T cell therapy

CAR-T cell immunotherapy achieves precise targeting and killing of tumor cells through genetic engineering modifications. However, its efficacy as a monotherapy is frequently limited by antigen escape, immunosuppression in the tumor microenvironment, and insufficient cell persistence in vivo. Consequently, combination strategies have emerged as pivotal approaches to enhance therapeutic efficacy [339, 340]. Dual-target CAR-T combination therapy effectively reduces the risk of antigen escape and significantly improves long-term survival outcomes in patients with hematologic malignancies. For instance, a five-year follow-up study of acute lymphoblastic leukemia patients demonstrated that sequential allogeneic hematopoietic stem cell transplantation followed by dual-targeted CD19 and CD22 chimeric antigen receptor T-cell therapy achieved a 75% overall survival rate and a 50% event-free survival rate, with markedly improved long-term survival outcomes [341]. The combination of CAR-T therapy and radiotherapy, chemotherapy, or immune checkpoint inhibitors can reshape the tumor microenvironment, enhance CAR-T cell infiltration and activation, and expand its application prospects in solid tumors [342–345]. Furthermore, optimizing CAR-T cell function and enhancing cytokine-targeted delivery efficiency through CRISPR gene editing can further improve treatment specificity and safety [346, 347].

In virus-associated tumors, CAR-T cell targets primarily focus on viral-encoded proteins (e.g., HPV E6/E7, EBV LMP2A) and host tumor-associated antigens (e.g., GPC3, desmoglein 18.2) [347, 348]. Additionally, molecules such as epidermal growth factor receptor, CD70, and mucin 1 have been identified as potential targets for chimeric antigen receptor T-cell immunotherapy [349, 350]. Similarly, Guo et al. developed the antibody 2H5-A14 targeting the pre-S1 region of the HBV large envelope protein and engineered A14 CAR-T cells. Their study demonstrated that these cells effectively cleared HBV-infected hepatocytes while inducing proinflammatory and antiviral cytokine production [351]. Wu et al. further confirmed the significant therapeutic efficacy of chimeric antigen receptor T-cell immunotherapy in hepatocellular carcinoma [352].

Currently, CAR-T combination therapy has demonstrated significant efficacy in certain solid tumors and hematologic virus-associated malignancies. However, its application in most virus-related tumors (such as HTLV-1 and KSHV) remains exploratory. Future research should focus on integrating chimeric antigen receptor T-cell immunotherapy with immune checkpoint blockade therapy, oncolytic virus therapy, and other modalities to leverage the advantages of different treatment approaches and enhance therapeutic outcomes for virus-associated cancers.

#### RNA-based combination therapies

RNA therapeutics can exert effects by targeting viral antigens or oncogenic genes. Mainstream RNA therapeutic agents encompass multiple technologies including messenger RNA (mRNA) vaccines, small interfering RNA (siRNA), microRNA (miRNA) modulators, antisense oligonucleotides (ASO), RNA aptamers, and the CRISPR/Cas9 gene editing system. Their combination with conventional therapies or immunotherapy can enhance treatment efficacy for virus-associated tumors through multi-target synergistic effects.

Among these, personalized mRNA vaccines—which leverage their favorable safety profile and rapid development timeline—markedly potentiate specific T-cell responses in conjunction with immune checkpoint inhibitors and have exhibited efficacy across a range of tumor entities [353–356]. For instance, Lin and colleagues engineered a self-amplifying mRNA (saRNA) vaccine built upon an adenoviral backbone. This construct incorporates the granulocyte–macrophage colony-stimulating factor-E7 (GM-CSF-E7) immunostimulatory factor, which not only enhances T-cell effector function, inhibits cervical cancer cell growth, and prolongs mouse survival, but also demonstrates significant advantages over traditional mRNA vaccines [357]. In models of EBV-associated nasopharyngeal carcinoma, researchers combined mRNA vaccines with natural killer (NK) cell therapy, enhancing treatment efficacy in mouse models and laying the groundwork for subsequent clinical trials [358]. Additional viral targets remain under active investigation for mRNA-based therapeutic strategies [359–362]. siRNA and miRNA modulators regulate tumor-associated signaling pathways through gene silencing mechanisms, enhancing tumor cell sensitivity to chemotherapy or immunotherapy [363, 364]. One study combined stereotactic body radiotherapy (SBRT) with miR-21-targeted therapy, improving radiotherapy efficacy in a lung cancer mouse model [365].

ASOs and CRISPR/Cas9 systems offer novel pathways to overcome drug-resistant tumors by precisely modulating gene expression or achieving permanent gene knockout, demonstrating particular advantages in targeting genes associated with viral latent infection (e.g., HBV cccDNA, EBV EBNA1) [366–369]. Additionally, researchers used ASOs to knock out SET-and-MYND-domain-containing protein 3 (SMYD3), which epigenetically suppresses the expression of type I interferon response genes in HPV-negative head and neck squamous cell carcinoma (HNSCC) cells. In a mouse oral carcinoma (MOC1) model, SMYD3 knockout significantly enhanced tumor sensitivity to PD-1 therapy [370], suggesting that combining ASOs with PD-1 inhibitors may represent a potentially effective therapeutic pathway. Moreover, CRISPR/Cas9 technology has been applied to therapeutic research against the novel coronavirus (SARS-CoV-2). The Abbott team developed the preventive antiviral CRISPR system (PAC-MAN), which utilizes CRISPR-Cas13d to target highly conserved sequences within the SARS-CoV-2 genome, cleaving viral fragments in lung epithelial cells. However, its efficacy and safety remain to be validated [371].

RNA aptamers exert targeted suppression by binding with high affinity to tumor cell surface markers. Combining them with other RNA therapies or chemotherapeutic agents can further enhance treatment specificity [372–374]. Additionally, Ji et al. developed the G4-SLSELEX-Seq platform targeting G4 motifs, integrating a pre-designed stem-loop structure library into the SELEX screening process. Using this platform, researchers identified the aptamer L-Apt1-12, which effectively downregulates EBNA1 expression in EBV-infected cancer cells and exhibits selective cytotoxicity toward EBV-positive cancer cells. Concurrently, they screened an L-type RNA aptamer targeting the rG4 structure of hepatitis C virus 1a (HCV-1a) [375].

Despite the promising prospects of combined RNA therapy strategies, common challenges persist, including low in vivo delivery efficiency, poor tissue targeting, and unvalidated long-term safety. These represent core directions for future clinical translation research. Future studies addressing these issues may provide novel directions and therapeutic approaches for treating virus-associated cancers.

#### Combination therapies with oncolytic viruses

Combining traditional chemotherapy and radiotherapy with oncolytic viruses (OV) can enhance antitumor immune responses. Moreover, the combination of oncolytic viruses with other immunotherapies can also produce synergistic antitumor effects. Notably, the combination of oncolytic viruses with ICIs has been demonstrated in clinical and preclinical studies to significantly enhance antitumor immune activity. For example, treatment with the oncolytic adenovirus oAd-shCD24 combined with a PD-1 inhibitor showed significantly superior efficacy compared to monotherapy [376]. Oncolytic viruses can also synergize with CAR-T cell therapy by reshaping the TME to enhance CAR-T cell infiltration and function. For instance, oncolytic viruses expressing cytokines such as interleukin-12 (IL-12) or chemokine CCL5 recruit CAR-T cells to tumor sites, thereby improving therapeutic outcomes [377]. Zhang et al. developed an oncolytic virus expressing HPV immunogens. Their therapeutic strategy involved first inducing systemic T-cell heterologous priming via mRNA vaccines, then directing these activated T cells to tumor sites using the oncolytic virus, ultimately achieving significant tumor regression [378]. Li et al. engineered a novel oncolytic adenovirus, Ad-TBZ, which exhibits potent and specific antitumor activity against EBV-associated gastric cancer through selective replication mediated by the human telomerase reverse transcriptase promoter (hTERTp) and viral lytic reactivation induced by the EBV lytic gene BZLF1. However, studies indicate only moderate synergistic effects when this virus is combined with chemotherapy [379]. Currently, research on combinatorial oncolytic virus therapies targeting virus-associated cancers such as KSHV, HBV, HCV, MCPyV, HTLV-1, and HIV remains extremely limited.

Currently, oncolytic viruses are mainly administered to patients with advanced, refractory, or recurrent malignancies that have progressed despite standard interventions—including surgery, chemoradiation, targeted therapy, and immunotherapy [380]. Clinical trial results confirm their significant efficacy. In a representative study, Shen and colleagues achieved a disease control rate (DCR) of 64.86% and an objective response rate (ORR) of 18.92% using genetically engineered oncolytic herpes simplex virus (VG161) to treat refractory hepatocellular carcinoma patients. Patients demonstrated a progression-free survival (PFS) of 2.9 months and an overall survival (OS) of 12.4 months. Compared to patients previously treated with immune checkpoint inhibitors, this therapy doubled median survival (17.3 months vs. 7.4 months) with favorable safety profiles [380]. Zhong et al. developed another novel oncolytic virus therapy. They intravenously administered recombinant Newcastle disease virus (NDV-GT) carrying the porcine α1,3-galactosyltransferase (α1,3-GT) gene, masking tumors as porcine-like organs to trigger hyperacute rejection and induce the immune system to attack tumor tissue. This approach not only suppressed tumor growth but also achieved complete tumor clearance, demonstrating a disease control rate (DCR) of up to 90% in patients with recurrent/refractory metastatic cancer, with some achieving complete remission [381]. Additionally, a clinical study investigated the efficacy of the oncolytic virus T-VEC combined with chemotherapy or endocrine therapy in patients with hormone receptor-positive (HR⁺)/human epidermal growth factor receptor 2-negative (HER2⁻), triple-negative advanced breast cancer with injectable paracranial/chest wall lesions. These results revealed a partial response rate (PR) of 12.5% and a disease stabilization rate of 43.8% [382], f thereby underscoring the distinct advantages of combining oncolytic viruses with conventional treatment modalities.

## Emerging technologies and research progress

**Fig. 5:**
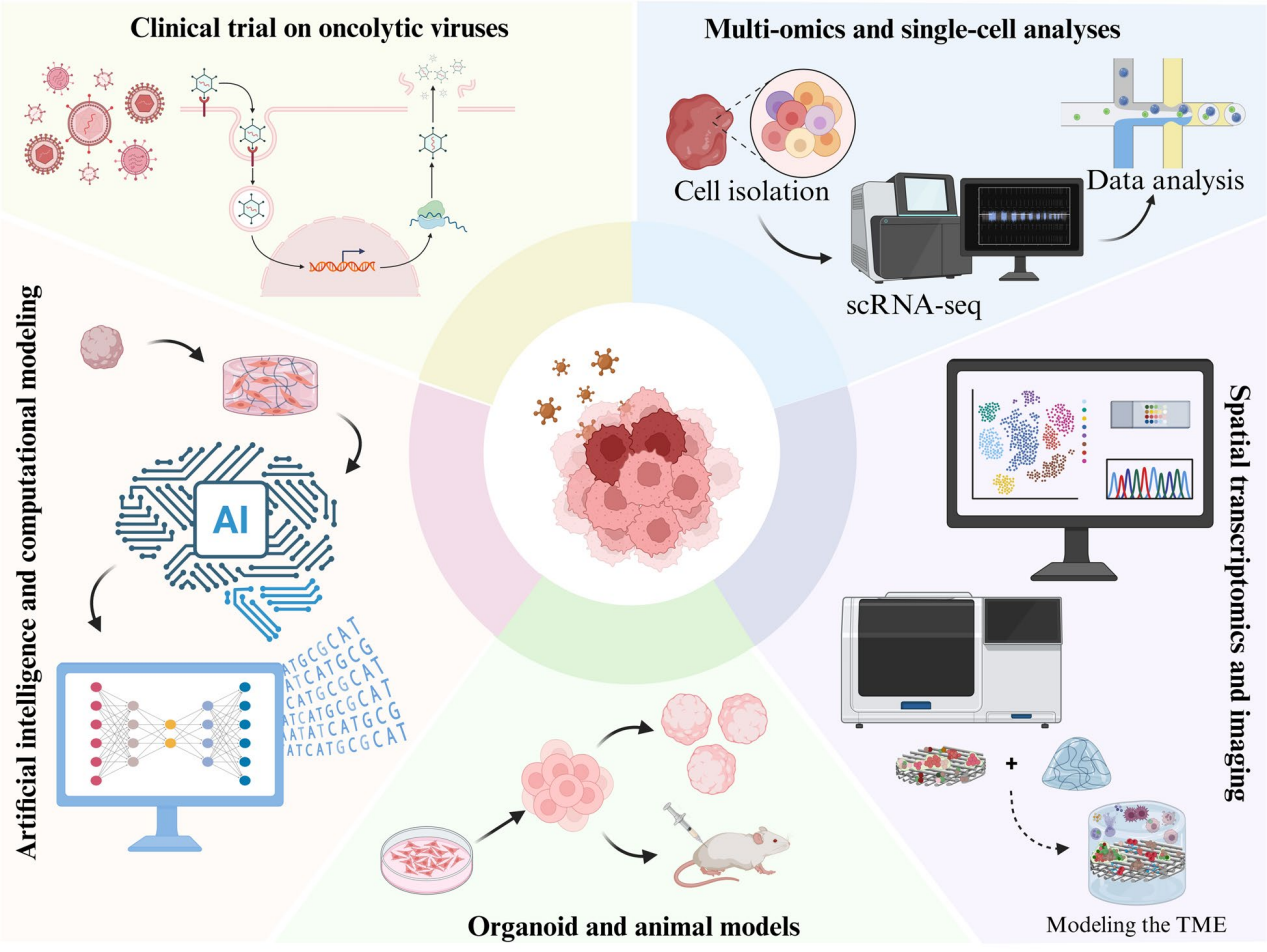
Emerging technologies in virus-induced carcinogenesis research. The mechanisms by which viral infections induce cancer remain incompletely understood, and traditional research approaches are no longer sufficient. Integrating emerging technologies with conventional methods will significantly advance our understanding of viral carcinogenesis. (Top right) Multi-omics and single-cell analyses: scRNA-seq enables analysis of viral RNA transcription, viral integration sites, and host gene expression profiles at the single-cell level. Multi-omics analysis integrates data from different levels of biological systems, comprehensively revealing the interaction networks between viruses and hosts at the systems level. (Bottom right) Spatial transcriptomics and imaging: Combining high-resolution imaging with spatial sequencing technologies enables the mapping of gene expression patterns while preserving the tissue's native spatial structure, thereby revealing the spatial heterogeneity of the tumor microenvironment. (Center bottom) Organoids and animal models: Utilizing organoids and animal models as research platforms simulating virus-driven tumorigenesis, these approaches construct tumor microenvironments to recreate the complex pathological processes of virus-induced carcinogenesis and test therapeutic responses. (Bottom left) Artificial intelligence and computational modeling: Applying AI algorithms, neural networks, and bioinformatics tools to process complex genomic sequence data, constructing virtual experimental platforms and prognostic models for viral carcinogenesis, providing scientific basis for personalized treatment of virus-associated malignancies. (Top left) Clinical trial on oncolytic viruses: Oncolytic virus therapy is a novel immunotherapy that harnesses viruses to attack cancer cells. Genetically engineered viruses gain the ability to replicate selectively within tumor cells, causing tumor lysis and activating the host's anti-tumor immune response

The field of viral oncology is undergoing a revolution driven by cutting-edge technologies, fueled by the convergence of multidisciplinary fields including molecular biology, genomics, and computational biology. Virus-cancer research is advancing rapidly with unprecedented depth and breadth. The application of emerging technologies has not only significantly deepened our understanding of viral carcinogenesis mechanisms but also opened new pathways for developing more precise and efficient cancer diagnosis and treatment strategies. The integrated application of multi-omics technologies, single-cell analysis, spatial transcriptomics, organoids, animal models, and artificial intelligence is progressively unraveling the intricate mechanisms of virus-host interactions and accelerating the clinical translation of therapeutic strategies (Fig. 5).

### Multi-omics and single-cell analyses to dissect virus–cancer interactions

Traditional virus-cancer research has primarily focused on individual genes or proteins. However, viral infection is a complex process involving dynamic changes across multiple levels, including the viral genome, host transcriptome, proteome, and metabolome. Regarding the core questions of target cell types, cellular heterogeneity, and interaction networks in viral infection, breakthrough insights have been achieved through the synergistic application of single-cell RNA sequencing (scRNA-seq) with conventional transcriptomics and multi-omics technologies (genomics, transcriptomics, metabolomics, etc.). Multomics technologies reveal the global reshaping of host molecular networks triggered by viral infection, while scRNA-seq captures gene expression dynamics and intercellular communication features at single-cell resolution, overcoming limitations of traditional sequencing [383, 384]. The table below summarizes key molecular features, cellular interactions, and potential therapeutic targets of major oncogenic viruses across conventional, single-cell, and spatial resolution omics studies (Table 4).

**Table 4:** Primary carcinogenic viruses in conventional transcriptome, single-cell transcriptome, and spatial transcriptome studies

Virus	Conventional Transcriptomics/Multi-Omics	Single-Cell Transcriptomics	Spatial Transcriptomics/Imaging Technologies	Ref
HPV	4-EA promotes cancer (metabolic marker); Integration affects methylation & expression	Identified immune signatures of HPV ±; LAG3 as a potential target	SPP1 + macrophages shape immunosuppressive microenvironment; Depict immune cell dynamics	[385–390]
EBV	LMP1 drives glycolytic reprogramming; Reprogramming GPCRome promotes NKTCL progression	Tumor cells have dual characteristics; Discovered CLEC9A DC subset	Tertiary lymphoid structure key cells determine immunotherapy efficacy	[224, 391–393]
HBV	GWAS identified genetic risk loci; Drives lipid metabolic reprogramming; Discovered host factors for vaccine production	Revealed SPP1-CD44 interaction axis; SLC16A3 and other prognostic genes; MMP9 promotes metastasis	Revealed integration sites and antiviral treatment effects	[394–400]
KSHV	Discovered novel transcriptional subtypes and transcription start sites	To be investigated	To be investigated	[401]
HCV	Metabolomic and lipidomic profiles of HCC show superior prediction accuracy over AFP	Depicted myeloid cell atlas before/after cure, found ISG, PD-L1/L2, etc., changes in specific cell types	To be investigated	[402, 403]
MCPyV	81.8% found viral integration into human genome	To be investigated	MCC spontaneous regression correlates with virus positivity & presence of tertiary lymphoid structures	[404, 405]
HTLV-1	FOXO3, ANKRD11, DGKZ, and PTPN6 may be potential tumor suppressors	p16C protein may be a key leukemogenic factor	To be investigated	[406, 407]
HIV	To be investigated	IL1B associated with latent reservoir; Viral control correlates with infected cell clearance	vRNA assembly function; Visualization of early infection	[408–411]
SARS-CoV-2	To be investigated	m⁶A regulator IGF2BP2 impairs lung regeneration	To be investigated	[412]

4-EA, 4-Ethylamino; AFP, Alpha-Fetoprotein; ANKRD11, Ankyrin Repeat Domain 11; CLEC9A, C-Type Lectin Domain Containing 9 A; DC, Dendritic Cell; DGKZ, Diacylglycerol Kinase Zeta; FOXO3, Forkhead Box O3; GPCRome, G Protein-Coupled Receptor repertoire; GWAS, Genome-Wide Association Study; IGF2BP2,: Insulin-like Growth Factor 2 mRNA-Binding Protein 2; IL1B, Interleukin-1 Beta; ISG, Interferon-Stimulated Gene; LAG3, Lymphocyte-Activation Gene 3; LMP1, Latent Membrane Protein 1; m^6^A, N6-methyladenosine; MCC, Merkel Cell Carcinoma; MMP9, Matrix Metalloproteinase 9; NKTCL, Natural Killer/T-Cell Lymphoma; PD-L1/L2, Programmed Death-Ligand 1/2; PTPN6, Protein Tyrosine Phosphatase Non-Receptor Type 6; SLC16A3, Solute Carrier Family 16 Member 3; SPP1, Secreted Phosphoprotein 1; vRNA, Viral RNA

The combined application of multi-omics and single-cell sequencing technologies is further advancing research into viral carcinogenesis mechanisms. Although challenges remain—including the complexity of integrating multi-omics data, the high cost of single-cell sequencing, and the intricacy of data interpretation—the integration of these multi-techniques with emerging technologies like AI algorithms promises to reconstruct the dynamic process from viral infection to tumorigenesis more comprehensively and precisely. This approach provides robust technical support for elucidating the pathogenesis of virus-associated cancers and identifying therapeutic targets.

### Spatial transcriptomics and imaging for viral tumor ecosystems

Spatial transcriptomics and high-resolution imaging techniques—such as multiplex immunofluorescence and photoactivated localization microscopy (PALM) —have played a crucial role in elucidating the tissue-spatial localization of virus-infected cells and their local microenvironment interaction patterns. Spatial transcriptomics enables high-throughput detection of gene expression profiles while preserving tissue architecture, precisely mapping the spatial distribution of infected cells, immune cells, and stromal cells. High-resolution imaging techniques achieve subcellular localization of molecular markers. Integrating spatial transcriptomics gene expression data with protein expression information from multicolor immunofluorescence (mIF) reveals how viral integration upregulates epithelial-mesenchymal transition (EMT)-related gene expression, visually demonstrating how infected cells remodel their surrounding microenvironment [389, 413–415] (Table. 4).

While these techniques have achieved breakthroughs in deciphering the solid tumor microenvironment, their application in virology remains in its infancy. As these technologies continue to mature, they will play a pivotal role in elucidating viral pathogenic mechanisms, advancing drug development, and optimizing diagnostic strategies, thereby providing a solid foundation for the prevention and control of viral carcinogenesis.

### Organoid and animal models of virus-driven tumorigenesis

Organoids, as three-dimensional in vitro models derived from adult stem cells or tumor tissues, can highly mimic in vivo tissue structures and physiological functions. Genetically engineered animal models, meanwhile, can reproduce the complete pathological process of viral carcinogenesis (including immune evasion and distant metastasis) to enable in vivo validation [416].

For instance, HPV-associated cervical cancer organoids can closely simulate tumorigenic potential and chemotherapy resistance in a clinically relevant manner [417]; Patient-derived organoids (PDOs) from HPV-associated head and neck squamous cell carcinoma precisely reproduce tumor pathological features, mutation profiles, and drug response characteristics [418], offering significant advantages for therapeutic development. Additionally, McCallister's team constructed KSHV organoids supporting cellular transformation following viral infection [419], while marmot liver virus (WHV)-infected marmots provide a natural model for HBV-related hepatocellular carcinoma research [420]. The MCPyV-associated SLAP mouse model drives tumorigenesis by inducing viral T antigen expression, Atoh1 expression, and p53 deficiency [421]. while adult T-cell leukemia virus type 1 (HTLV-1) transgenic mice spontaneously develop lymphoproliferative disorders [422].

These models provide reliable platforms for personalized drug screening and in vivo validation of targets. However, existing organoids lack key components such as immune cells and neural cells, and animal models struggle to fully replicate the dynamics of natural viral infection [416]. With the integrated application of relevant technologies, it is anticipated that more ideal models closely resembling human tumors and their microenvironments will be developed in the future.

### Artificial intelligence and computational modeling in viral oncology

Faced with the challenge of interpreting massive multi-omics, imaging, and clinical data, AI and computational modeling techniques (such as convolutional neural networks (CNN), random survival forests (RSF), and gradient boosting machines (GBM)) have achieved automation and precision in biomarker screening, diagnostic classification, and prognostic assessment by integrating multidimensional data [423, 424].

For instance, in the context of diagnostic classification, the Roche team employed a CNN model to differentiate between normal and tumor organoids derived from head and neck squamous cell carcinoma [418]. By integrating computed tomography (CT) data, the nnU-Net model enabled automated classification of extranodal invasion in patients with HPV-positive cN + oropharyngeal carcinoma, yielding superior accuracy relative to manual assessment [425]. The YOLOv5 and ST3DCN models have respectively enhanced the efficiency of ultrasound detection for liver lesions and classification of CT nodules [426, 427]. These technological advances provide crucial support for early screening and management of hepatocellular carcinoma. Furthermore, in therapeutic applications, AI-driven drug target screening platforms identified significant upregulation (p < 0.01) of both transcriptional and translational levels of the CACNA2D1 gene in nasopharyngeal carcinoma tissues, suggesting its potential as a therapeutic target [428]. Cryo-electron microscopy (cryoEM) and cryo-electron tomography (cryoET) technologies, combined with AI, successfully resolved the three-dimensional structures of EBV and KSHV particles. This identified unique features of the viral envelope glycoproteins and outer nucleocapsid layers, laying the foundation for studying their mechanisms of human infection [429]. In the field of prognostic prediction, Farzaneh's team employed advanced machine learning models such as support vector machines (SVM) and neural networks (NN) to enhance the accuracy of predicting the severity of HPV-induced cervical intraepithelial neoplasia (CIN) [430]. A GBM-based model (PLAN-B) can predict hepatocellular carcinoma risk in chronic HBV carriers [431]. Additionally, the Nanbo team developed the SMART model using the RSF algorithm to assess hepatocellular carcinoma risk after HCV cure [432]. Collectively, these technologies provide crucial support for early screening, treatment decisions, and personalized medicine.

These computational tools not only accelerate biomarker discovery but also provide data-driven support for personalized treatment decisions in virus-associated cancers. By constructing virus-host interaction networks, they elucidate the complex mechanisms of viral carcinogenesis at the systems level, laying the foundation for understanding virus-induced tumorigenesis and progression mechanisms as well as prognostic inference.

### Clinical trial on oncolytic viruses

OVs represent a novel immunotherapy strategy engineered to selectively replicate within tumor cells, lysing the tumor while releasing tumor-associated antigens to activate anti-tumor immunity, thereby achieving a dual effect of “tumor lysis + immune activation” [433].

For instance, therapeutic mRNA vaccines face limitations in overcoming the immunosuppressive barriers of the tumor microenvironment for HPV-associated cancers. In response, Zhang et al. developed a type 1 herpes simplex virus (HSV-1) oncolytic virus expressing HPV immunogens and combined it with mRNA vaccines, achieving significant tumor regression [378]. Additionally, the recombinant oncolytic virus SONC103 specifically cleaves the HPV16 oncogene and restore p53/pRB function, thereby inhibiting tumor cell proliferation and promoting apoptosis. It demonstrated significant tumor regression in mouse models [434]. Furthermore, the oncolytic adenovirus Ad-TBZ can upregulate the expression of EBV lytic genes, increase viral genome copy numbers, and induce cell line-specific late apoptosis, significantly enhancing the efficacy against EBV-associated gastric cancer [379]. Furthermore, an adenoviral therapeutic system targeting KSHV(AAV8-TR2-OriP-TK) combined with ganciclovir achieves precise clearance of KSHV-positive cells, demonstrating promising antitumor and antiviral effects in preclinical models [435]. However, oncolytic viruses still face numerous challenges in clinical translation. These include limitations in delivery methods confined to intratumoral injection, making it difficult to effectively target deep-seated tumors or metastatic lesions; low viral replication efficiency in mouse models that fails to fully mimic human responses; difficulties in balancing antiviral and anti-tumor immune regulation; lack of predictive biomarkers for efficacy; and potential safety concerns [436, 437].

Oncolytic virus therapy has emerged as a research hotspot in viral oncology, opening novel pathways for treating virus-associated cancers and a spectrum of solid tumors. It offers new therapeutic hope for advanced cancer patients unresponsive to conventional treatments. Additionally, research on the tumor cell microbiome is gaining momentum. Analysis of microbial sequences in single-cell RNA sequencing data by Robinson's team revealed the coexistence of different microbial genera within host cells in esophageal and colorectal cancer samples. This observation suggests potential interactions among multiple intracellular microbes [438]. Similar studies indicate that microbes within pancreatic tumors may be associated with cellular motility and immune signaling pathways, confirming that tumor-microbiome interactions may participate in regulating tumorigenesis and progression [439]. These findings hold promise for guiding the development of immunotherapy strategies based on tumor-associated microbial characteristics, driving precision cancer diagnosis and treatment toward greater refinement.

## Conclusion

Although current research has clarified the epidemiological characteristics of classic oncogenic viruses such as HPV and EBV and their pivotal roles in tumorigenesis, the carcinogenic potential of emerging viruses remains understudied. Preliminary research indicates that emerging viruses like SARS-CoV-2 may indirectly promote tumor progression by inducing chronic inflammation and disrupting host immune homeostasis [118]. However, the dose–response relationship between viral infection and tumorigenesis, the temporal window effects, and the synergistic mechanisms when combined with other carcinogenic factors (such as smoking or genetic susceptibility loci) remain incompletely elucidated. This research gap limits our comprehensive understanding of viral carcinogenesis and poses challenges for developing tumor prevention strategies. Therefore, future large-scale cohort studies combined with multi-omics analyses are essential to elucidate the carcinogenic mechanisms and key regulatory nodes of emerging viruses.

Although prior work has characterized several discrete layers of viral oncogenesis—including oncogene activation, genomic integration, and epigenetic regulation—the crosstalk between different mechanisms and their stage-specific roles in tumor progression remain to be further investigated. HPV and HBV demonstrate vaccine-based cancer prevention strategies. HCV highlights how curative antiviral therapy may function as cancer prevention. EBV and KSHV underscore the importance of latency, immune modulation, and stromal remodeling. HIV and HTLV-1 show that virus-associated carcinogenesis may also arise through systemic or compartment-specific disruption of immune homeostasis rather than through direct cellular transformation alone. Emerging viral threats, including SARS-CoV-2 and other newly recognized pathogens, further broaden this perspective by suggesting that long-term oncologic consequences may also emerge from chronic inflammatory or immune-dysregulatory states even in the absence of classical transforming viral behavior.

Moreover, molecular-level viral carcinogenesis does not occur in isolation but is closely intertwined with the dynamic remodeling of TME. The carcinogenic “niche” formed by chronic inflammation and fibrosis serves as a critical bridge linking viral infection to tumor progression. However, how viruses precisely regulate interactions between inflammatory cells and stromal cells to maintain niche stability remains to be explored. For instance, HCV infection induces activation of hepatic stellate cells, leading to excessive extracellular matrix secretion and the formation of a fibrotic microenvironment that promotes hepatocellular carcinoma. However, the paracrine signaling pathways between viral proteins (e.g., Core protein) and hepatic stellate cells/macrophages, along with the regulatory roles of inflammatory mediators (e.g., TNF-α, IL-6), require further refinement. Concurrently, the co-evolutionary relationship between virus-induced immune exhaustion mechanisms and the inflammatory “niche” remains unclear. Organoid models can simulate the dynamic changes in TME following viral infection, potentially providing experimental evidence for optimizing combined therapeutic strategies.

These insights have important translational implications. Future progress in virus-associated cancers will depend not only on continued characterization of viral oncogenes and host pathways, but also on integrated strategies that connect pathogen control with cancer prevention, early detection, immune restoration, and microenvironment-directed therapy. In practice, the most effective interventions are likely to combine vaccination, antiviral treatment, biomarker-driven surveillance, immune modulation, and therapies targeting stromal and metabolic vulnerabilities, particularly in settings where delayed diagnosis, unequal healthcare access, and persistent co-infection continue to shape outcomes.

Looking ahead, further advances in the field will depend on technologies that resolve virus–host interactions with greater precision, including multi-omics integration, single-cell and spatial transcriptomics, organoid systems, artificial intelligence-assisted risk prediction, and improved longitudinal population studies. These approaches will help clarify why common infections lead to cancer only in selected individuals, how viral persistence drives tissue-specific oncogenesis, and which molecular or microenvironmental dependencies can be exploited therapeutically. Overall, virus-associated cancers should be viewed not as isolated entities defined solely by pathogen name, but as dynamic biological systems in which infection, host response, and tissue ecology co-evolve over time. A clearer understanding of this integrated landscape will be essential for more effective prevention, mechanistic insight, and precision treatment in the coming years.

## Data Availability

Not applicable.

## Abbreviation

HPV: Human papillomavirus
EBV: Epstein-Barr virus
KSHV: Kaposi's sarcoma-associated herpesvirus
HHV-8: Human herpesvirus 8
KS: Kaposi’s sarcoma
HBV: Hepatitis B virus
HCV: Hepatitis C virus
MCPyV: Merkel cell polyomavirus
MCC: Merkel cell carcinoma
HTLV-1: Human T-lymphotropic virus type 1
HIV: Human immunodeficiency virus
HPyV: Human polyomavirus
SARS-CoV-2: Severe acute respiratory syndrome coronavirus 2
CHIKV: Chikungunya virus
TME: Tumor microenvironment
ICIs: Immune checkpoint inhibitors
LMP1: Latent membrane protein 1
LANA: Latency-associated nuclear antigen
ECM: Extracellular matrix
E6/E7: HPV E6/E7 oncoproteins
NF-κB: Nuclear factor kappa-light-chain-enhancer of activated B cells
STAT3: Signal transducer and activator of transcription 3
TGF-β: Transforming growth factor-beta
HIF-1: Hypoxia-inducible factor 1
PIK3: Phosphatidylinositol 3-kinase
ROS: Reactive oxygen species
CAD: Carbamoyl-phosphate synthetase 2, aspartate transcarbamoylase, and dihydroorotase
GLUT1: Glucose transporter 1
ATLL: Adult T-cell leukemia/lymphoma
scRNA-seq: Single-cell RNA sequencing
CAFs: Cancer-associated fibroblasts
OV: Oncolytic virus
MAPK: Mitogen-activated protein kinase
EndMT: Endothelial-mesenchymal transition
FIB-4: Fibrosis-4 index
HBZ: Human T-cell leukemia virus type I bZIP factor
EMT: Epithelial-mesenchymal transition
Treg: Regulatory T cell
CIN: Cervical intraepithelial neoplasia
XGBoost: Extreme gradient boosting
LR: Logistic regression
CNN: Convolutional neural network
RF: Random forest
SVM: Support vector machine
GBM: Gradient boosting machine
RSF: Random survival forest
PD-1: Programmed death-1
NPC: Nasopharyngeal carcinoma
LT-ag: Large tumor antigen
sT-ag: Small tumor antigen
VEGF: Vascular endothelial growth factor
gp350/370: Glycoprotein 350/370
gH/gL/gB: Glycoprotein H/L/B
MDSC: Myeloid-derived suppressor cells
vGPCR: Viral G protein-coupled receptor
IARC: International agency for research on cancer
PEL: Primary exudative lymphoma
MCD: Multicentric Castleman disease
HR: Hazard ratio
